# Characterization of the Metabolic Requirements in Yeast Meiosis

**DOI:** 10.1371/journal.pone.0063707

**Published:** 2013-05-08

**Authors:** Debjit Ray, Ping Ye

**Affiliations:** 1 School of Molecular Biosciences, Washington State University, Pullman, Washington, United States of America; 2 Biological Systems Engineering, Washington State University, Pullman, Washington, United States of America; 3 Center for Reproductive Biology, Washington State University, Pullman, Washington, United States of America; Tata Institute of Fundamental Research, India

## Abstract

The diploid yeast *Saccharomyces cerevisiae* undergoes mitosis in glucose-rich medium but enters meiosis in acetate sporulation medium. The transition from mitosis to meiosis involves a remarkable adaptation of the metabolic machinery to the changing environment to meet new energy and biosynthesis requirements. Biochemical studies indicate that five metabolic pathways are active at different stages of sporulation: glutamate formation, tricarboxylic acid cycle, glyoxylate cycle, gluconeogenesis, and glycogenolysis. A dynamic synthesis of macromolecules, including nucleotides, amino acids, and lipids, is also observed. However, the metabolic requirements of sporulating cells are poorly understood. In this study, we apply flux balance analyses to uncover optimal principles driving the operation of metabolic networks over the entire period of sporulation. A meiosis-specific metabolic network is constructed, and flux distribution is simulated using ten objective functions combined with time-course expression-based reaction constraints. By systematically evaluating the correlation between computational and experimental fluxes on pathways and macromolecule syntheses, the metabolic requirements of cells are determined: sporulation requires maximization of ATP production and macromolecule syntheses in the early phase followed by maximization of carbohydrate breakdown and minimization of ATP production in the middle and late stages. Our computational models are validated by *in silico* deletion of enzymes known to be essential for sporulation. Finally, the models are used to predict novel metabolic genes required for sporulation. This study indicates that yeast cells have distinct metabolic requirements at different phases of meiosis, which may reflect regulation that realizes the optimal outcome of sporulation. Our meiosis-specific network models provide a framework for an in-depth understanding of the roles of enzymes and reactions, and may open new avenues for engineering metabolic pathways to improve sporulation efficiency.

## Introduction

Meiosis is a strongly conserved cell division program that generates haploid gametes from a diploid parental cell. Successful meiosis is the fundamental basis of sexual reproduction. Meiosis has been studied extensively in multiple model systems, ranging from the budding yeast *Saccharomyces cerevisiae* to the mouse. Multiple lines of evidence suggest a tight link between meiosis and metabolism [1]. In yeast, meiosis is promoted by nutrient deprivation [2], while in the mouse, both male and female germ cells enter meiosis in response to retinoic acid [3], [4]. In the nematode *C. elegans* and the fruit fly *Drosophila melanogaster*, ablation of the germline leads to profound alternations in lipid metabolism [5], [6]. We focus on yeast meiosis in this study to elucidate the link between reproduction and metabolism, which could improve our understanding of metabolic control on the meiotic process.

The diploid yeast undergoes mitosis in glucose-rich medium. When cells are transferred to acetate sporulation medium, a developmental switch from mitosis to meiosis occurs to generate four haploid spores [2]. This switch depends on the heterozygous mating-type locus as well as the deprivation of a fermentable carbon source and nitrogen [7]. The yeast metabolic machinery has to make a remarkable adaptation to environmental signals by sequentially activating or inhibiting a large number of enzymes in different pathways. This adaptation ensures the cell will meet its energy and biosynthetic requirements during sporulation. Therefore, the metabolic network is crucial in determining the success of yeast meiosis.

**Figure 1:**
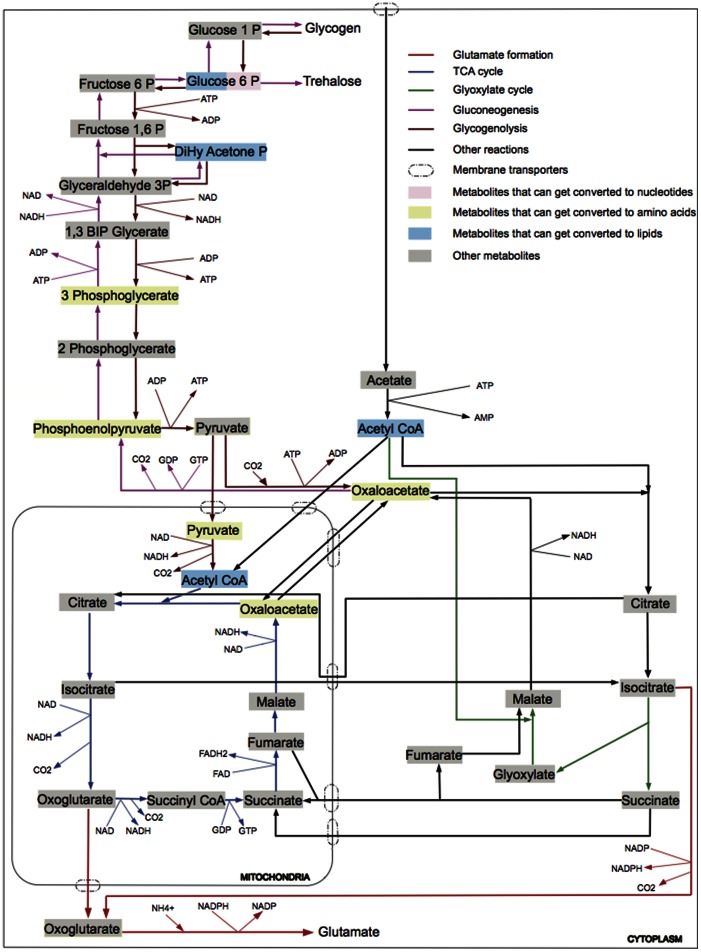
A meiosis-specific metabolic network in yeast. Five metabolic pathways are active during meiosis when acetate serves as the external carbon source: glutamate formation, TCA cycle, glyoxylate cycle, gluconeogenesis, and glycogenolysis. Metabolites in these pathways are utilized to synthesize macromolecules: nucleotides, amino acids, and lipids.

Conspicuous differences have been observed in metabolism between mitosis and meiosis. In mitosis, glucose is consumed by fermentation or respiration via the tricarboxylic acid (TCA) cycle. In meiosis, acetate serves as the sole carbon source and five metabolic pathways are active at different stages: glutamate formation, TCA cycle, glyoxylate cycle, gluconeogenesis, and glycogenolysis (Figure 1) [8], [9], [10], [11], [12], [13], [14], [15]. The slow assimilation of acetate during meiosis as compared to the rapid consumption of glucose in mitosis is believed to allow the adaptation from glycolysis to gluconeogenesis and avoid depletion of intracellular ATPs [14]. During meiosis, external acetate is first converted to acetyl-CoA and metabolized by respiration via the TCA cycle to produce energy [9], [10], [14]. Glutamate is first generated from the precursor oxoglutarate, which is an intermediate from the TCA cycle [9]. The reaction of glutamate formation removes ammonium ion, a strong inhibitor of yeast sporulation [16]. The glyoxylate cycle is concurrently active with the TCA cycle to replenish TCA intermediates that are exhausted due to the production of glutamate [9]. Gluconeogenesis involves the synthesis of carbohydrates in the form of trehalose and glycogen, which account for two-thirds of the mass increase during meiosis [11]. Notably, glutamate production ceases when the gluconeogenesis pathway becomes fully functional [9]. The glyoxylate cycle also occurs concurrently with gluconeogenesis to supply substrates [14]. During spore maturation, acetate has been exhausted from the medium [9], [10], [14]; glycogen is then consumed through glycogenolysis to provide energy [8]. The sequential activation of metabolic pathways and the interplay between these pathways supply cells with the required energy and macromolecules, i.e., nucleotides, amino acids, and lipids [9], [10], [13], [15].

Although the pathways are well characterized from biochemical studies, the optimal principles responsible for the dramatic metabolic changes accompanying meiosis are poorly understood. The cellular objectives, i.e., the metabolic requirements of cells, are undetermined. We construct a meiosis-specific metabolic network in yeast and apply a constraint-based modeling approach—flux balance analysis (FBA) [17]—to characterize the metabolic requirements of sporulating cells. FBA predicts flux distribution in a metabolic network using linear or nonlinear programming with the knowledge of reaction stoichiometry, objective functions, and reaction constraints. The advantages of FBA are that it circumvents the need for enzyme kinetic parameters that are difficult to measure, can be easily scaled up for investigating genome-scale networks, and is well suited for characterizing *in silico* perturbations such as gene or reaction knockout [18]. FBA has been used with great success in studying organism or tissue-specific metabolism with applications such as engineering pathways [19], [20], [21], [22], [23], [24], [25], [26], [27]. Although several genome-scale metabolic networks have been published and FBA approaches have been applied to investigate yeast metabolism, these studies mainly focus on unlimited mitotic growth of cells on glucose [21], [26], [28], [29], [30]. Our study serves as the first attempt to characterize a meiosis-specific metabolic network in yeast by FBA. Using time-series gene expression as reaction constraints, we identify the dynamic metabolic requirements of cells at different phases of meiosis. Our network models provide a framework to predict novel enzymes essential for yeast meiosis, some of which have been verified in the literature. Ultimately, this study elucidates the optimal principles driving the operation of yeast metabolic network, which ensure the successful completion of sporulation.

## Results

### The dynamic profile of a yeast meiosis-specific metabolic network

A meiosis-specific metabolic network is reconstructed based on the literature and databases [28], [29], [31], [32], [33], [34], [35], [36], [37], [38], [39], [40], [41], [42], [43], [44], [45], [46], [47], [48], [49], [50], [51], [52], [53], [54]. This manually curated network comprises 31 metabolites and 62 reactions catalyzed by 69 enzymes (Figure 1, Table S1, Model S1). Five pathways are described when utilizing external acetate as the sole carbon source: glutamate formation, TCA cycle, glyoxylate cycle, gluconeogenesis, and glycogenolysis. Macromolecules produced from these pathways include nucleotides, amino acids, and lipids. This meiosis-specific network is better suited to study yeast meiosis for several reasons: 1) it includes reactions and enzymes specific for acetate metabolism; 2) it incorporates reactions and enzymes that are missing in genome-scale reconstructions; and 3) every reaction has a known enzyme-reaction association. A detailed comparison between the meiosis-specific network and iMM904 [28], a genome-scale yeast metabolic network, is shown in Table S2.

**Figure 2:**
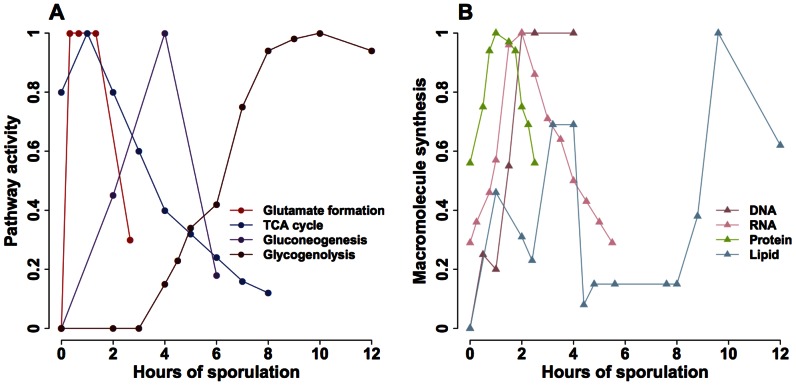
Scaled biochemical data on metabolic pathways and macromolecule syntheses during yeast meiosis. The time scale of sporulation (12 hours) is defined by the SK1 strain. Datasets obtained using other strains are standardized to the SK1 time scale based on the duration when the ascus level reaches a steady state. Activities of metabolic pathways and macromolecule syntheses are further scaled to the range of 0 and 1. Raw and scaled biochemical data are summarized in Table S3. **A.** Pathway activity: glutamate formation [9], [10], TCA cycle [13], gluconeogenesis [11], and glycogenolysis [8]. **B.** Macromolecule synthesis: DNA [13], RNA [13], protein [13], and lipid [15].

However, metabolic pathways and macromolecule syntheses are not constitutively active throughout meiosis. Instead, each has a unique dynamic profile based on experimental studies [8], [9], [10], [11], [12], [13], [14], [15], [55]. We analyze in parallel several biochemical datasets (Table S3). Because experimental methods used for measuring pathways and macromolecules are different, it is not possible to directly compare their activities. For example, the activity of the TCA cycle is estimated based on oxygen consumption, whereas glycogenolysis activity is determined by enzyme kinetics [8], 13. Therefore, each pathway activity is scaled to the range of 0 and 1 to observe the dynamics throughout meiosis. In addition, different yeast strains are used in these biochemical experiments. Thus, data obtained using other strains are calibrated to the time scale of SK1, a strain commonly used for studying meiosis, according to the duration when ascus formation reaches a steady state. The scaled pathway activity and the scaled sporulation time are shown in Figure 2; we find a sequential activation of pathways and macromolecule syntheses. Glutamate is the major metabolite produced during the first four hours of sporulation [9], [10]. The activity of the TCA cycle peaks at two hours and subsequently declines [13], [14]. Gluconeogenesis, including the syntheses of glycogen and trehalose, rises later and reaches the highest level at four hours of sporulation [8], [9], [11], [12], [13]. In contrast, glycogenolysis does not initiate until four hours and is the only active pathway during the late stage of meiosis [8], [13]. Although no experimental data is available for the glyoxylate cycle, its activity can be estimated from glutamate formation and gluconeogenesis. The glyoxylate cycle supplies TCA cycle intermediates for glutamate formation and gluconeogenesis during early and mid-phase of meiosis, showing correlated activity with these two pathways [9], [14]. DNA, RNA, and proteins are synthesized early during the first four hours of sporulation [10], [13], whereas lipid production peaks at four and ten hours, respectively [10], [15].

### Metabolic requirements of yeast cells during sporulation

To determine the metabolic requirements of sporulating yeast, we systematically evaluate ten objective functions relevant to meiosis: ATP production, ATP consumption, net ATP production (production minus consumption), acetate uptake, glutamate synthesis, carbohydrate synthesis, carbohydrate breakdown, nucleotide synthesis, amino acid synthesis, and lipid synthesis. Not all enzymes are expressed at any given time during meiosis. To reflect the dynamic profile of the metabolic network, we constrain the upper bound of reactions using time-course ribosome profiling data on SK1 sporulating cells [56]. Ribosome profiling measures ribosome-protected mRNA levels by deep sequencing, which approximate to enzyme activities. The absolute gene expression data are used to create continuous, rather than discrete reaction bounds. A total of 18 time points spanning 12 hours of sporulation are considered.

**Figure 3:**
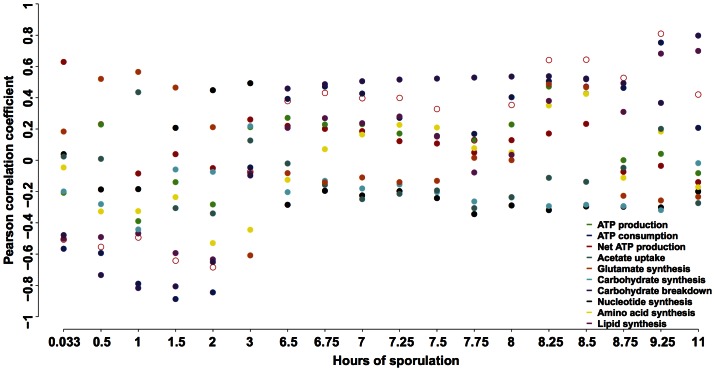
Evaluation of objective functions using the meiosis-specific network models. The Pearson correlation is calculated between predicted fluxes and biochemical data on eight pathways when maximizing or minimizing each of the ten objective functions at each of the 18 time points. The best objective function for each time point is the one with the maximum Pearson correlation coefficient. Close circle: maximization of an objective function; open circle: minimization of an objective function. Undefined correlation coefficients due to zero variance of predicted pathway fluxes are not shown in the figure.

The ten objective functions are individually maximized or minimized at each of the 18 time points; this is equivalent to evaluating 360 FBA models. The optimal solution of network fluxes is determined for each model. We calculate the Pearson correlation between predicted fluxes and biochemical data (Table S4) on eight pathways and macromolecule syntheses: glutamate formation, TCA cycle, glyoxylate cycle, gluconeogenesis, glycogenolysis, nucleotides, amino acids, and lipids (Table S1, abbreviated as eight pathways in the rest of the paper). The Pearson correlation measures a linear relationship between computational and biochemical data when simultaneously considering multiple pathways. It allows the identification of the most relevant objective function based on quantitative metrics. Because pathway activity and macromolecule synthesis exhibit distinct dynamic patterns, the metabolic requirements of cells must change. Indeed, different best objective functions, i.e., the objective with the maximum correlation coefficient at each time point, are identified throughout the period of sporulation (Figure 3).

At 0.033 hour immediately after yeast cells are transferred to the acetate medium, net ATP production maximization is the best objective function. When using expression data at 0.5, 1, and 1.5 hours as reaction constraints, the metabolic requirement is best described by glutamate synthesis maximization. This is in accordance with the report that glutamate is the major metabolite produced early from acetate utilization [9]. At 2 and 3 hours, nucleotide synthesis maximization becomes the most suitable objective function, consistent with the experimental observation that DNA and RNA syntheses reach the maximum at that time [10], [13]. From 6.5 to 8 hours as well as at 11 hours, carbohydrate breakdown maximization best describes the cellular objective; biochemical data show that glycogenolysis is the only pathway active in the late stage of meiosis [8], [13]. From 8.25 to 9.25 hours, net ATP production minimization is the best objective function. Yeast cells are moving towards a dormant phase; most pathways except glycogenolysis are turned off and lipids are the only macromolecule being synthesized [8], [10], [13], [15].

Although the solution space has been constrained by network structures, reaction stoichiometry, reaction bounds, and objective functions, linear optimization often leads to multiple optimal flux distributions with an identical objective value. To determine whether the optimal solution identified using our method is an artifact of one single optimum or a representative of multiple optima, we sample the solution space of meiosis-specific network models for each objective function while constraining the objective value to its optimum. The sampling is performed at each time point during sporulation using expression-based constraints. We find that alternative optima exist for carbohydrate breakdown maximization (6.5, 7, 7.25, 7.75, 8, 8.25, 8.75, and 9.25 hours) and acetate uptake maximization (8, 8.25, and 8.75 hours), both having only one reaction in the objective function. Pearson correlations are calculated between alternative optima and biochemical data on pathways to obtain the distribution of correlations at each time point. The correlation value of optimum identified by our method falls within the range of sampled correlations for carbohydrate breakdown maximization at all eight time points (Figure S1) and for acetate uptake maximization at one out of three time points, suggesting that most optimal solutions identified using our method are consistent with multiple optima derived from sampling the solution space. The lack of multiple optima for other objective functions may be explained by the relatively small size of the meiosis-specific network.

To provide further proof of best objective functions identified along the time series, we implement four alternative methods to deduce objectives. First, instead of using the Pearson correlation, we calculate the Spearman correlation between predicted fluxes and biochemical data. All best objective functions except those during 6.5–8 hours reproduce as using the Pearson correlation (Figure S2). Second, instead of using gene expression to constrain the maximum flux through reactions, we apply iMAT [20] to obtain context-specific networks through maximizing the consistency between reaction fluxes and gene expression states. Sixteen out of 18 networks capture reactions that participate in the best objective functions determined by the Pearson correlation (Table S5). Third, instead of using expression data from ribosome profiling, we apply microarray data at 1, 2, 3, 4, 6, 8, and 10 hours of sporulation [55] to constrain the maximum flux through reactions. All best objective functions except those at 6 and 8 hours adhere to the objectives deduced from ribosome profiling (Figure S3). Finally, instead of using gene expression to constrain the maximum flux through reactions, we impose a uniform constraint on all reactions. Only three out of 18 best objective functions reproduce as those identified using gene expression, indicating the importance of incorporating expression data to deduce meiosis-specific metabolic requirements (Figure S4). Results from the first three methods further justify the sequential change of objective functions during sporulation, as determined by constraining reactions with ribosome profiling data and calculating the Pearson correlation between predicted fluxes and biochemical data.

### Model validation using known sporulation-deficient genes

We use independent data—data distinct from those used for model building—to evaluate the meiosis-specific network models. High-throughput screens of ∼4,000 yeast deletion strains have identified 267 genes required for sporulation, i.e., sporulation-deficient genes, as well as 102 genes that enhance sporulation proficiency [57]. Our meiosis-specific network contains 16 of the sporulation-deficient genes but none of the sporulation-enhanced genes, suggesting the network includes essential reactions of yeast meiosis. The 16 enzymes catalyze 13 reactions, most of which are in TCA cycle, while others participate in glyoxylate cycle, gluconeogenesis, acetate uptake, and amino acid synthesis. Each of the 16 enzymes catalyzes only one reaction, while some reactions are catalyzed by more than one of those enzymes.

**Figure 4:**
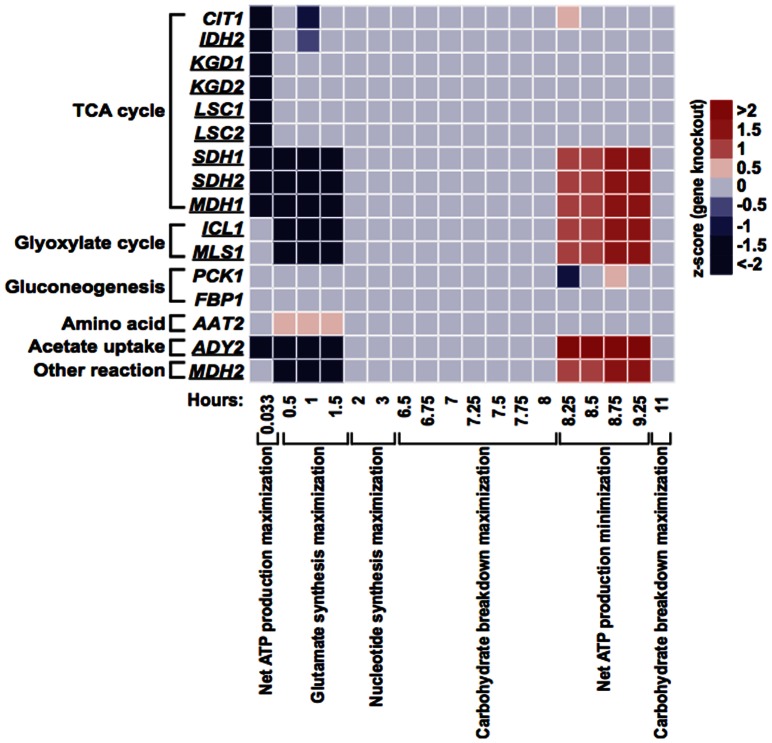
Validation of the meiosis-specific network models using known sporulation-deficient genes. A total of 16 enzymes in the meiosis-specific network are known to be essential for sporulation. These genes are individually deleted *in silico*; optimal fluxes are obtained using the best objective function combined with expression-based constraints at each time point. Pearson correlations are calculated between optimal fluxes and biochemical data on eight pathways for gene KOs. Deviation from the WT correlation is quantified by a z-score. Gene KOs with a z-score ≤−2 for at least one time point are considered to validate the models (underlined).

To verify the meiosis-specific network models, we delete each of the 16 sporulation-deficient genes *in silico* and obtain the optimal fluxes for a gene knockout (KO) using the identified best objective function and expression-based constraints at every time point. The Pearson correlation between *in silico* fluxes of a KO and biochemical data on eight pathways is calculated in a similar way as that for wild-type (WT). The KO effect is quantified by a z-score that measures the difference in correlation coefficient between a KO and WT. This allows us to assess each KO's change in correlation with experimental values in units of standard deviation, a form of the z test. A negative z-score indicates that the KO causes pathway fluxes less correlated with biochemical data compared to WT for a given time point. Using the criterion of having a z-score less than -2 for at least one time point, our models validate 12 out of 16 sporulation-deficient genes (Figure 4).

All 12 validated KOs exhibit reduced correlation with biochemically determined pathway data during the first 1.5 hours of sporulation when net ATP production maximization and glutamate synthesis maximization are the most relevant objective functions. These genes have essential functions in TCA cycle (*IDH2*, *KGD1*, *KGD2*, *LSC1*, *LSC2*, *SDH1*, *SDH2*, *MDH1*), glyoxylate cycle (*ICL1*, *MLS1*), acetate uptake (*ADY2*), and others (*MDH2*), thus affecting sporulation at an early stage.

**Figure 5:**
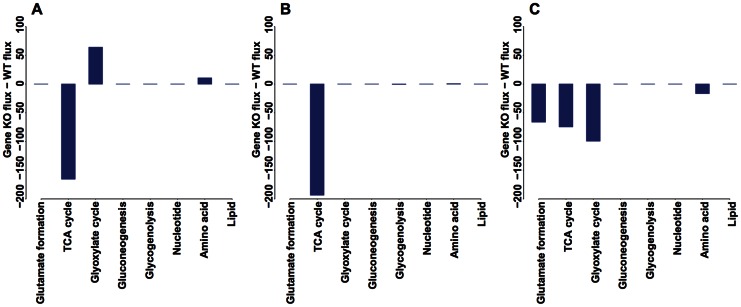
Flux changes in metabolic pathways and macromolecule syntheses when individually deleting known sporulation-deficient genes. **A**. *IDH2* deletion at 0.033 hour. Single gene KOs of *KGD1*, *KGD2*, *LSC1*, and *LSC2* show similar flux changes at the same time. **B**. *SDH1* deletion at 0.033 hour. Single gene KOs of *SDH2*, *MDH1*, and *ADY2* show similar flux changes at the same time. **C**. *ICL1* deletion at 1.5 hours. Single gene KOs of *SDH1*, *SDH2*, *MDH1*, *MLS1*, *ADY2*, *MDH2*, and *ICL1* show similar flux changes at 0.5, 1, and 1.5 hours.

We further examine the flux difference between validated KOs and WT on eight pathways (Figure 5). At 0.033 hour, when net ATP production maximization is the best objective function, KOs exhibit either a reduced flux in TCA cycle alone (*SDH1*, *SDH2*, *MDH1*, *ADY2*) or in combination with an increased flux in glyoxylate cycle (*IDH2*, *KGD1*, *KGD2*, *LSC1*, *LSC2*). Between 0.5 and 1.5 hours, when glutamate synthesis maximization is the most favored objective, reduced flux is observed in glutamate formation, TCA cycle, and glyoxylate cycle (*SDH1*, *SDH2*, *MDH1*, *ICL1*, *MLS1*, *ADY2*, *MDH2*).

### Model prediction of novel genes required for sporulation

**Figure 6:**
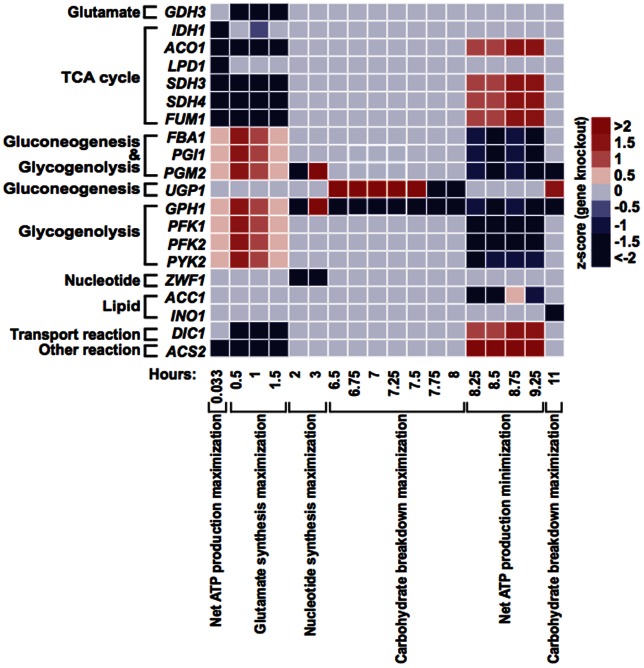
Model prediction of novel genes required for sporulation. Every gene in the meiosis-specific network is deleted *in silico*; optimal fluxes are obtained using the best objective function combined with expression-based constraints at each time point. Pearson correlations are calculated between optimal fluxes and biochemical data on eight pathways for gene KOs. Deviation from the WT correlation is quantified by a z-score. Genes previously unknown to be required for sporulation and having a z-score ≤−2 for at least one time point are predicted to be novel sporulation-deficient genes.

High-throughput screens of deletion strains have discovered more than 300 genes required for sporulation, many are associated with metabolic pathways [57], [58], [59], [60]. However, we hypothesize that not all sporulation-deficient genes have been revealed through these approaches. To predict novel metabolic genes essential for yeast meiosis, we delete every gene in the meiosis-specific network *in silico* and find 32 out of 69 KOs are predicted to affect sporulation as defined by having a z-score less than −2 for at least one time point, the same criterion used for the validation experiment. Among the 32, 12 are known sporulation-deficient genes and the remaining 20 are potential novel genes required for meiosis (Figure 6).

Deletions of each of the 20 genes disrupt network fluxes at different phases of sporulation. In fact, these genes are identified when pathways that they belong to become the most relevant objective function at the time. At 0.033 hour, when net ATP production maximization is the best objective function, additional genes in the TCA cycle (*IDH1*, *ACO1*, *LPD1*, *SDH3*, *SDH4*, *FUM1*) and acetate transport (*ACS2*) are predicted to be required for sporulation. These KOs result in reduced TCA activity or both enhanced glyoxylate cycle flux and reduced TCA activity, as we have seen in deletions of known sporulation-deficient genes (Figure 5A–5B). When the metabolic requirement is best described as glutamate synthesis maximization at 0.5–1.5 hours, deleting genes in glutamate formation (*GDH3*), TCA cycle (*ACO1*, *SDH3*, *SDH4*, *FUM1*, *DIC1*), and acetate metabolism (*ACS2*) cause reduced correlation of predicted pathway fluxes with experimentally determined values. In these KOs, *in silico* fluxes in glutamate formation, TCA cycle, and glyoxylate cycle are reduced compared to WT, similar to the pattern seen for the KOs of known sporulation-deficient genes at the same time frame (Figure 5C).

In contrast to known sporulation-deficient genes that impact *in silico* fluxes only during the first 1.5 hours, 11 out of 20 predicted novel genes are detected after two hours of sporulation. At 2–3 hours, when nucleotide synthesis maximization is the most relevant objective function, deletion of *PGM2*, *GPH1*, or *ZWF1* results in significantly reduced correlation between experimental and computational fluxes on pathways. *ZWF1* controls the initial step of nucleotide synthesis from glucose-6-phosphate, while *PGM2* and *GPH1* catalyze reactions producing glucose-6-phosphate. A common feature of *in silico* fluxes in these three KOs is a dramatic reduction of nucleotide synthesis. From 6.5 to 8 hours of sporulation, when the metabolic requirement is best described as carbohydrate breakdown maximization, *GPH1* is consistently predicted to affect sporulation. *GPH1* mediates the first reaction of glycogenolysis—glycogen breakdown. Thus, it is unsurprising the *GPH1* KO exhibits a reduced flux in glycogenolysis. When sporulation reaches the end stage from 8.25 to 9.25 hours, net ATP production minimization becomes the most suitable objective function. Eight enzymes are predicted to be essential for sporulation at that time frame; all except *ACC1* participate in glycogenolysis (*FBA1*, *PGI1*, *PGM2*, *GPH1*, *PFK1*, *PFK2*, *PYK2*). *ACC1* converts cytoplasmic acetyl-CoA to lipids; the *ACC1* KO exhibits reduced fluxes in lipid synthesis. Deletions of the other seven genes lower fluxes in both glycogenolysis and lipid synthesis. At 11 hours of sporulation, carbohydrate breakdown maximization becomes the best objective function again. *PGM2*, *GPH1*, and *INO1* are predicted to be required for sporulation. *INO1* converts glucose-6-phosphate into lipids, while *PGM2* and *GPH1* catalyze initial reactions in glycogenolysis to produce glucose-6-phosphate. These gene KOs completely shut down the lipid synthesis and decrease the glycogenolysis activity.

### Literature validation of predicted sporulation-deficient genes

**Figure 7:**
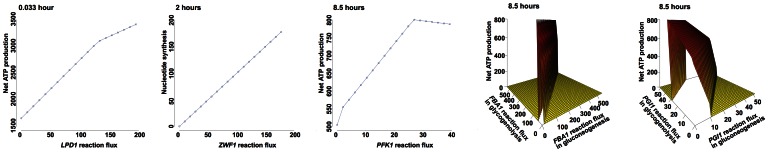
Robustness analyses on reactions catalyzed by predicted genes required for sporulation. The objective function value is computed as the flux through the reactions varies. The best objective function combined with expression-based constraints at a specific time is used for the robustness analysis.

The meiosis-specific network models predict 20 genes that potentially affect sporulation, among which four are essential for cell viability (*SDH3*, *UGP1*, *ACC1*, *ACS2*) [61]. Literature studies of the other 16 genes reveal that five are, indeed, required for sporulation (*LPD1*, *ZWF1*, *PFK1*, *FBA1*, *PGI1*) [62], [63], [64], [65]; deletion of *GPH1* still permits sporulation [52]. Although no sporulation-deficiency phenotype has been reported for the remaining 10 genes, they are promising candidates for future experimental testing. We perform a robustness analysis on reactions catalyzed by the five literature-validated genes (Figure 7). The robustness analysis allows the computation of how the value of an objective function changes as the flux along one or multiple reactions varies in magnitude, revealing the sensitivity of the objective to reactions [66].

*LPD1* encodes dihydrolipoamide dehydrogenase, an enzyme required for TCA cycle in the mitochondria. Homozygous *lpd1* diploids are unable to sporulate, suggesting the enzymatic activity is essential [63]. *LPD1* is predicted to affect sporulation immediately after meiotic initiation (Figure 6). Robustness analysis indicates that net ATP production increases with flux through the *LPD1* reaction. A complete deletion of the reaction is predicted to lower the optimal level of ATPs by half (Figure 7).

*ZWF1* encodes a cytoplasmic glucose-6-phosphate dehydrogenase that catalyzes the first step of the pentose phosphate pathway. The pentose phosphate pathway is important for generating NADPH as well as a variety of sugar molecules that are required for the biosynthesis of nucleic acids. A strain homozygous for the *zwf1* mutation sporulates at a reduced level compared to WT [64]. In our meiosis-specific network, the *ZWF1* reaction is the only reaction controlling nucleotide synthesis and is predicted to affect sporulation during hours 2–3 when nucleotide synthesis maximization is the best objective function (Figure 6). Robustness analysis reveals a linear relationship between nucleotide production and the *ZWF1* reaction flux. Deletion of the *ZWF1* reaction is predicted to result in no nucleotide synthesis (Figure 7).

*PFK1* catalyzes the formation of fructose 1,6-bisphosphate from fructose 6-phosphate and ATP. Unlike other enzymes that function in both directions of gluconeogenesis and glycogenolysis, *PFK1* is specific to glycogenolysis and is required for sporulation [65]. Robustness analysis indicates that net ATP production is sustained near the optimal value over a range of flux values, demonstrating network robustness with respect to the flux change in the *PFK1* reaction. However, once the flux value drops below 27, ATP production is sensitive to changes in the reaction flux, exhibiting a linear decline. A complete deletion of the reaction is predicted to reduce ATP production to 500 that cannot meet the energy requirement of cells at the late phase of sporulation (Figure 7).

Both *FBA1* and *PGI1* catalyze reversible reactions in gluconeogenesis and glycogenolysis. Homozygous diploids bearing either *fba1* or *pgi*1 mutations are asporogenous, indicating an absolute requirement for gluconeogenic and glycogenolytic events in sporulation [62], [64]. These two genes are predicted to be essential at a late stage when net ATP production minimization is the best objective function (Figure 6). For both genes, we vary two reversible reaction fluxes simultaneously to observe the effects on net ATP production. The results are plotted as a 3-D surface, revealing the interaction between two reversible reactions. When *FBA1* reaction fluxes are at their optimal values (gluconeogenesis reaction = 0, glycogenolysis reaction = 27), the maximum net ATP production is achieved at 800. *FBA1* KO results in the deletion of two reversible reactions, reducing the net ATP production to 500. With such amount of ATP production, the cell is unable to meet the energy requirement for viability. Similarly, *PGI1* KO decreases net ATP production from the maximum value to 500 that is predicted to be deleterious to cells at a late phase of sporulation.

### Metabolic requirements of sporulating yeast determined by a genome-scale metabolic network

To evaluate whether the identified metabolic requirements of sporulating yeast are network-dependent, we repeat the analysis using iMM904, a genome-scale yeast metabolic network consisting of 1,577 reactions catalyzed by 904 enzymes [28]. The definition for ten objective functions and eight pathways remains the same as that of the meiosis-specific network (Table S1). Pearson correlations are calculated between experimental and computational flux values on pathways when constraining the model with objective functions and expression-based reaction bounds. We again find that the metabolic requirements of yeast cells evolve during sporulation, starting from ATP production maximization during the first three hours and followed by (net) ATP production minimization throughout the rest period (Figure S5). Objective functions identified from the genome-scale network are overall consistent with those determined from the meiosis-specific network. Sporulating cells first maximize the production of energy and macromolecules by relying on acetate as the sole carbon source. Subsequently, under nutrient scarcity, cells reduce all pathway activities by limiting ATP production, leading to the dormant stage of spores. Results from the genome-scale network indicate that our findings on metabolic requirements of sporulating yeast are robust and independent of networks.

We compare the performance of meiosis-specific and genome-scale network models in predicting KO sporulation deficiency (Table 1). Among 69 genes in the meiosis-specific network, 16 are known sporulation-deficient genes and 32 are predicted to affect sporulation with a z-score less than −2 for at least one time point. Twelve predicted genes are in fact known sporulation-deficient genes, equivalent to a hypergeometric P-value of 0.009. The same analysis is conducted for iMM904: among 867 solvable KOs, 35 and 173 are known and predicted sporulation-deficient genes, respectively, and 13 predicted genes are true. Thus, the significance of iMM904 in detecting sporulation-deficient genes equals to 0.012, comparable to the meiosis-specific network. However, the precision of the meiosis-specific network in discovering known sporulation-deficient genes is higher than that of iMM904 (12/32 = 37.5% v.s. 13/173 = 7.5%).

**Table 1:** Performance comparison between the meiosis-specific network and iMM904 in predicting sporulation-deficient genes using hypergeometric P-values.

	meiosis-specific network	iMM904
z-score of Pearson correlations&	0.009	0.012
z-score of optimal objective values*	0.019	0.004
total flux differences from linear MOMA#	0.001	0.004

& The Pearson correlation between *in silico* fluxes and biochemical values on eight pathways is calculated for each gene KO and WT. A z-score is computed to measure the difference in correlation coefficient between a KO and WT. A KO with z-score≤−2 for at least one time point is predicted to be a sporulation-deficient gene.

* The optimal objective value is obtained for each gene KO and WT. A z-score is computed to measure the difference in optimal objective value between a KO and WT. A KO with z-score≤−2 for at least one time point is predicted to be a sporulation-deficient gene.

# The total flux difference between a gene KO and WT is obtained from linear MOMA. A KO with flux difference≥1000 for at least one time point is predicted to be a sporulation-deficient gene.

To further assess the performance of network models, we implement two alternative approaches to estimate KO effects (Table 1). The first is to compute the difference in optimal objective value between a KO and WT and then transform the difference into a standard z-score. The second is to compute the total flux difference between a KO and WT using linear MOMA [67], [68]. By calculating the hypergeometric P-values of KO effects, we find both alternative approaches achieve comparable performance to our method—z-score of Pearson correlations. Further, both methods support the finding that the significance level of detecting known sporulation-deficient genes is similar for meiosis-specific and genome-scale networks.

## Discussion

Yeast meiosis is a reproductive process producing four haploid spores from one diploid cell. Shifting from a mitotic to a meiotic state requires multiple adjustments within the cell. Indeed, cells constantly tailor their metabolic machinery to meet new energy and biosynthesis requirements, and this is no less true in the meiotic process. Decades of biochemical research have identified most enzymes that catalyze metabolic reactions. However, the optimal principles that drive the dynamic adaptation of metabolic pathways are poorly understood.

FBA can be utilized to predict flux distribution in a metabolic network when the objective function is known. Because metabolic requirements are uncharacterized in yeast meiosis, we test a total of ten probable objective functions to uncover the optimal principles for different phases of meiosis. Although FBA predicts flux distribution at a steady state, the use of time-course expression data for reaction bounds allows us to change constraints, generating dynamic fluxes over the entire period of yeast meiosis. The ribosome-protected mRNA levels measured by deep sequencing [56] are an approximation of enzyme activity, nevertheless they are superior to total mRNA levels estimated from microarray experiments [55]. A systematic analysis of the relationship between *in silico* fluxes and *in vivo* fluxes is performed using quantitative metrics—the Pearson correlation coefficient. It measures the overall agreement between computational and experimental pathway values and allows direct comparison of the performance of all objectives. Our results from the meiosis-specific network suggest that the metabolic requirements of cells evolve during sporulation, starting with ATP production maximization followed by glutamate and nucleotide synthesis maximization. Carbohydrates in the form of glycogen are subsequently broken down to supply energy. At the end, under nutrient scarcity, cells reduce all pathway activities by limiting ATP production, which leads to the dormant stage of spores. Similar objective functions are determined from a genome-scale network: ATP production maximization at the early phase and ATP production minimization at the late stage. Consistent metabolic requirements revealed by two networks of different scales suggest the robustness of optimal flux solutions on pathways. These best objective functions reflect the cellular adaptation to the acetate environment by switching from glycolysis to gluconeogenesis, using ATP efficiently, and producing macromolecules for spore formation.

The meiosis-specific network model is validated using genes known to be required for sporulation [57] by studying the effects of single gene KOs. The model predicts the sporulation-deficiency phenotype with a significant hypergeometric P-value of 0.009, suggesting that the network specifically and accurately captures reactions active during yeast meiosis. The genome-scale models achieve a comparable P-value but with low precision. The advantage of genome-scale networks is that they contain all reactions occurring in a cell, including pathways that may become active when the meiosis process is under internal or external perturbations. However, genome-scale models have missing reactions and enzymes. For example, one third of reactions in iMM904 have no enzyme association; the average of genome-wide expression is used to constrain the maximum flux through these reactions, which may generate incorrect flux solutions. Predictions from genome-scale models will become more accurate with continued refinements of the reconstructions, e.g., adding gene-reaction associations.

The meiosis-specific network model is key to manipulating reactions or genes to determine their contribution to cell metabolism. We delete each gene in the network to study the effects of knockouts. These *in silico* perturbations pinpoint specific enzymes essential for sporulation, allowing for the prediction of novel sporulation-deficient genes that have not been identified by high-throughput screens [57], [58], [59], [60]. We have used quantitative metrics—the z-score—to prioritize predicted enzymes and verified some candidates based on the literature [52], [62], [63], [64], [65].

In summary, this study provides an in-depth knowledge of metabolic mechanisms during yeast meiosis. Further, the meiosis-specific network model offers a theoretical framework to investigate the contribution of enzymes and reactions to the sporulation phenotype. The model is powerful with respect to performing *in silico* experiments on any reaction or multiple reactions simultaneously, or on over-expression, knockdown, or knockout of any enzyme or combinations of multiple enzymes. Such analyses will provide insight into the metabolic machinery of yeast meiosis, and may open new avenues for engineering pathways to increase sporulation efficiency.

## Materials and Methods

### Construction of a meiosis-specific metabolic network in yeast

We manually construct a meiosis-specific metabolic network by including reactions known to be active during yeast meiosis. The network comprises pathways involved in the uptake of acetate from the external environment; the use of acetate for synthesizing carbohydrates, amino acids, nucleotides, and lipids; and, later, the breakdown of stored carbohydrates [8], [9], [10], [11], [12], [13], [14], [15]. Reactions and associated enzymes are obtained from the KEGG database, the *Saccharomyces* Genome Database, iMM904, and Yeast4.0 [28], [29], [31], [32]. Reactions are compartmentalized in either the cytoplasm or the mitochondria, based on the sub-cellular localization of enzymes determined by experimental evidence [33], [34], [35], [36], [37], [38], [39], [40], [41], [42], [43], [44], [45], [46], [47], [48], [49], [50], [51], [52], [53], [54]. Transport reactions across cellular or mitochondrial membrane are included in the network as well. All reactions are unidirectional; a reversible reaction is represented as two unidirectional reactions. Cofactors and end products are not included as metabolites in the network: ATP, ADP, AMP, GTP, GDP, UTP, UDP, NAD, NADH, NADP, NADPH, FAD, FADH2, CO2, NH4, H2O, glutamate, glycogen, trehalose, nucleotide, amino acid, and lipid. The resulting meiosis-specific metabolic network comprises 31 unique metabolites and 62 unique biochemical reactions catalyzed by 69 unique enzymes (Table S1, Model S1). The network in the Systems Biology Markup Language format is available at the BioModels database (http://www.ebi.ac.uk/biomodels/, MODEL1303140001).

We also investigate a genome-scale metabolic network–iMM904, which contains 1,228 metabolites and 1,577 reactions catalyzed by 904 unique enzymes [28]. To simulate the meiotic condition using iMM904, we allow the import of acetate and oxygen from the external environment but eliminate the intake of glucose and ammonium.

### Flux balance analysis

Flux is the rate at which metabolites are consumed or produced in a reaction. Flux in the metabolic networks of yeast meiosis is calculated using FBA at a steady state. Specially, all reactions in a metabolic network are mathematically represented as a stoichiometric matrix, 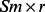, with *m* rows as metabolites and *r* columns as reactions. The entries in the matrix correspond to coefficients of metabolites for each reaction. Constraints are imposed for reactions: 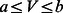, where *a* and *b* define the lower and upper bounds of allowable flux *V* for a reaction, respectively. An objective function defines a particular goal of cells, and can be represented as a linear combination of flux: 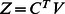 where *C* is a vector of weights on flux *V*. FBA seeks to maximize or minimize an objective function using linear programming by carrying out a steady-state analysis on 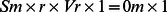. The outcome of FBA is one optimal flux distribution (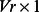, an optimal assignment of fluxes to all the reactions in the network) among many distributions that have the same optimal value for an objective function [18].

#### Objective functions

We systematically evaluate ten linear objective functions relevant to the metabolic requirements of cells during meiosis: 1) ATP production, 2) ATP consumption, 3) net ATP production, 4) acetate uptake, 5) glutamate synthesis, 6) carbohydrate synthesis, 7) carbohydrate breakdown, 8) nucleotide synthesis, 9) amino acid synthesis, and 10) lipid synthesis. Each objective function, 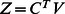, is maximized and minimized, respectively, where *C* is a vector of zeros with a value of one at the positions of reactions relevant to the objective function. The exceptions are three ATP objectives: positive numbers of yielded ATPs are coefficients for reactions participating in ATP production, positive numbers of used ATPs are coefficient for reactions participating in ATP consumption, and positive numbers of yielded ATPs and negative numbers of used ATPs are entered as coefficients for the objective function of net ATP production (Table S1).

#### Flux constraints

For the meiosis-specific metabolic network, the lower bound for all reactions is 0, implying irreversible reactions. The upper bound is determined by deep sequencing of ribosome-protected mRNA levels (GSE34082) [56]. A total of 18 time points are chosen to reflect the dynamic progression of meiotic events: 0.033, 0.5, 1, 1.5, 2, and 3 hours of sporulation from a traditionally-synchronized strain, and 6.5, 6.75, 7, 7.25, 7.5, 7.75, 8, 8.25, 8.5 8.75, 9.25, and 11 hours of sporulation from a Ndt80-inducible strain. The expression of 69 unique genes included in the meiosis-specific network is extracted from the dataset. For isozymes that each can individually catalyze the same reaction, the maximum of isozyme expressions is used as the reaction upper bound. For multi-unit enzymes or enzyme complexes that must act together to catalyze one reaction, the minimum of subunit expressions is used as the reaction upper bound (Table S1). Once upper bounds are determined for all reactions in the network based on enzyme expression, they are scaled to have an average value of 1,000 over the entire course of sporulation. For the iMM904 genome-scale model, the same procedure is applied to obtain the upper bounds for 1,577 reactions using the expression of 904 genes. The lower bounds of reversible reactions are given the negative of the upper bound values. For reactions with no enzyme association, the average of all upper bounds in iMM904 is used.

#### Computation

One optimal solution of network flux is obtained using GLPK (GNU Linear Programming Kit) supported by the COBRA Toolbox for Matlab [66]. Alternate optimal solutions are examined by random sampling the solution space using a hit-and-run algorithm—gpSampler [66]. For each objective function at each time point with expression-based constraints, we sample the solution space with 2,000 points for two minutes while constraining the objective value to its optimum. A mixed fraction within the range of 0.47–0.53 is used to ensure a proper sampling.

### Selection of the best objective functions

For each of the 18 time points, optimal solutions for network fluxes are obtained by maximizing or minimizing each of ten objective functions combined with expression-based reaction constraints. The Pearson correlation is calculated between predicted fluxes and biochemical data on eight pathways: 1) glutamate formation, 2) TCA cycle, 3) glyoxylate cycle, 4) gluconeogenesis, 5) glycogenolysis, 6) nucleotides, 7) amino acids, and 8) lipids. The Pearson correlation quantifies the linear relationship between two datasets and is defined as 
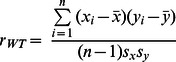
, where *x* and *y* are computational and experimental data, respectively, 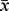 and 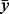
are means, 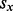 and 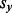 are standard deviations, and *n* is the number of pathways. Computational data on pathways are obtained by averaging optimal fluxes of reactions included in each pathway (Table S1). Biochemical data on pathways are obtained by linear interpolation of the scaled data to match the 18 time points (Table S4). Experimental values on glyoxylate cycle are the maximum of glutamate formation and gluconeogenesis at each time point. Experimental values on nucleotides are the maximum of DNA and RNA syntheses at each time point. The best objective function for each time point is selected as the one with the maximum Pearson correlation coefficient.

### Model prediction of sporulation-deficient genes

High-throughput screens of yeast deletion strains have identified 267 sporulation-deficient genes (pre-sporulation/sporulation≥1.5 in two independent experiments) [57]. For each sporulation-deficient gene included in a network, we delete the gene by changing the upper bound of the corresponding reaction and then calculate network fluxes. The Pearson correlation is computed between predicted KO fluxes and biochemical values on eight pathways, similar to the calculation for WT. Finally, a z-score is constructed to quantify the difference in the Pearson correlation between KO and WT: 
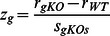
, where 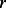 is correlation coefficient, 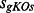 is the standard deviation of Pearson correlations of all gene KOs in the network. A z-score of less than -2 is considered as validating the sporulation-deficient phenotype.

### Performance evaluation of network models in predicting known sporulation-deficient genes

To determine whether known sporulation-deficient genes are enriched among model-predicted deficient genes, we calculate the P-value from a hypergeometric distribution: 
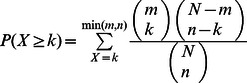
, where 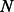
is the total number of genes in a metabolic network, 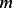 is the number of known sporulation-deficient genes in the network, 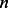 is the number of genes that the model predicts to affect sporulation, and 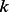 is the number of known sporulation-deficient genes predicted to affect sporulation as well.

## Supporting Information

Figure S1 **The optimum identified by our method for carbohydrate breakdown maximization is consistent with multiple optima derived from sampling the solution space of meiosis-specific network models.** For each of 18 time points with expression-based constraints, the sampling is performed with 2,000 points while constraining the objective value to its optimum. Multiple optimal solutions are identified for 8 time points. Pearson correlations are calculated between alternative optima and biochemical data on pathways. The quartiles of the distribution of correlations are displayed: maximum, 75 percentile, median, 25 percentile, and minimum. The Pearson coefficient calculated from one optimum, as shown in Figure 3, is displayed here again as dots.
(PDF)

Figure S2 **The use of Spearman correlation to evaluate objective functions for the meiosis-specific network models.** The Spearman correlation is calculated between predicted fluxes and biochemical data on eight pathways when maximizing or minimizing each of the ten objective functions at each of the 18 time points. The best objective function for each time point is the one with the maximum Spearman correlation coefficient. Eleven out of 18 best objective functions during 0.033–3 and 8.25–11 hours are consistent with those determined by the Pearson correlation as the first, second, or third ranked objective. Close circle: maximization of an objective function; open circle: minimization of an objective function. Undefined correlation coefficients due to zero variance of predicted pathway fluxes are not shown in the figure.
(PDF)

Figure S3 **The use of microarray data to evaluate objective functions for the meiosis-specific network models.** Reaction constraints are defined by gene expression from time-course *Affymetrix* data on SK1 sporulating cells at 1, 2, 3, 4, 6, 8, and 10 hours. The Pearson correlation is calculated between predicted fluxes and biochemical data on eight pathways when maximizing or minimizing each of the ten objective functions at each of the seven time points. The best objective function for each time point is the one with the maximum Pearson correlation coefficient. Five out of seven best objective functions at 1, 2, 3, 4, and 10 hours are consistent with those deduced from ribosome profiling. Close circle: maximization of an objective function; open circle: minimization of an objective function. Undefined correlation coefficients due to zero variance of predicted pathway fluxes are not shown in the figure.
(PDF)

Figure S4 **The use of uniform bounds to evaluate objective functions for the meiosis-specific network models.** A uniform constraint of 1,000 is imposed for all reactions. The Pearson correlation is calculated between predicted fluxes and biochemical data on eight pathways when maximizing or minimizing each of the ten objective functions at each of the 18 time points. The best objective function for each time point is the one with the maximum Pearson correlation coefficient. Three out of 18 best objective functions at 0.033, 2, and 3 hours are consistent with those deduced from ribosome profiling. Close circle: maximization of an objective function; open circle: minimization of an objective function. Undefined correlation coefficients due to zero variance of predicted pathway fluxes are not shown in the figure.
(PDF)

Figure S5 **Evaluation of objective functions using the genome-scale network models.** The Pearson correlation is calculated between predicted fluxes and biochemical data on eight pathways when maximizing or minimizing each of the ten objective functions at each of the 18 time points. The best objective function for each time point is the one with the maximum Pearson correlation coefficient. Close circle: maximization of an objective function; open circle: minimization of an objective function. Optimal solution does not exist regardless of objective functions at 2 hours of sporulation, thus does not shown in the figure.
(PDF)

Model S1 **The yeast meiosis-specific metabolic network in the Systems Biology Markup Language format.**
(XML)

Table S1 **Reactions in the yeast meiosis-specific metabolic network.**
(PDF)

Table S2 **A comparison between the meiosis-specific network and iMM904.**
(XLSX)

Table S3 **Biochemical data on metabolic pathways and macromolecule syntheses during yeast meiosis.**
(PDF)

Table S4 **Interpolated biochemical data on metabolic pathways and macromolecule syntheses at 18 time points.**
(PDF)

Table S5 **Context-specific networks determined by iMAT for incorporating expression data with the meiosis-specific network.**
(XLSX)
