# Supplementary figures and images for: Characterization of the Metabolic Requirements in Yeast Meiosis
Source: PLoS One. 2013 May 8;8(5):e63707. doi: 10.1371/journal.pone.0063707 (PMC3650881; doi:10.1371/journal.pone.0063707)

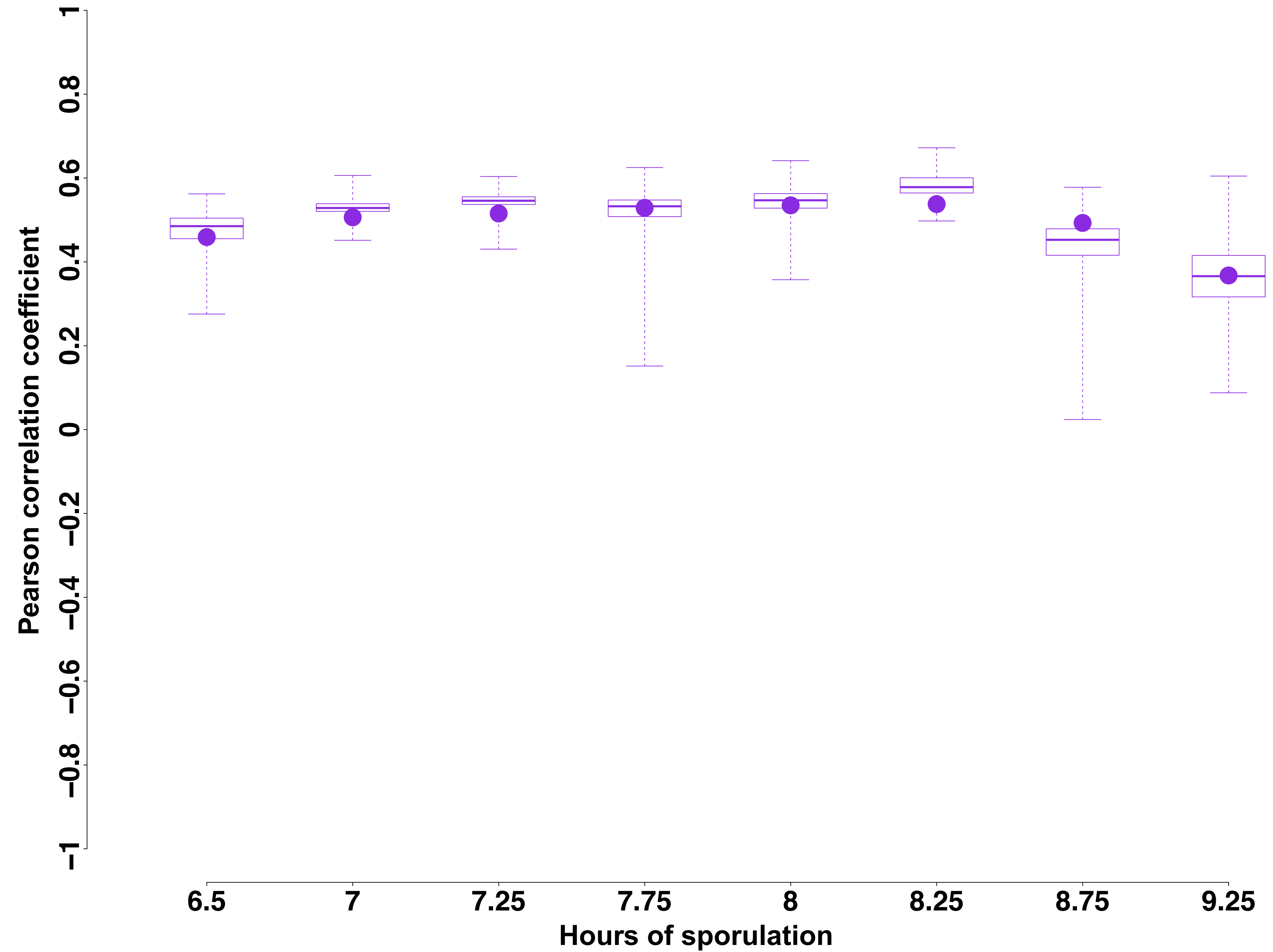

Supplement: Figure S1 — The optimum identified by our method for carbohydrate breakdown maximization is consistent with multiple optima derived from sampling the solution space of meiosis-specific network models. For each of 18 time points with expression-based constraints, the sampling is performed with 2,000 points while constraining the objective value to its optimum. Multiple optimal solutions are identified for 8 time points. Pearson correlations are calculated between alternative optima and biochemical data on pathways. The quartiles of the distribution of correlations are displayed: maximum, 75 percentile, median, 25 percentile, and minimum. The Pearson coefficient calculated from one optimum, as shown in Figure 3, is displayed here again as dots. (PDF) [file pone.0063707.s001.pdf]

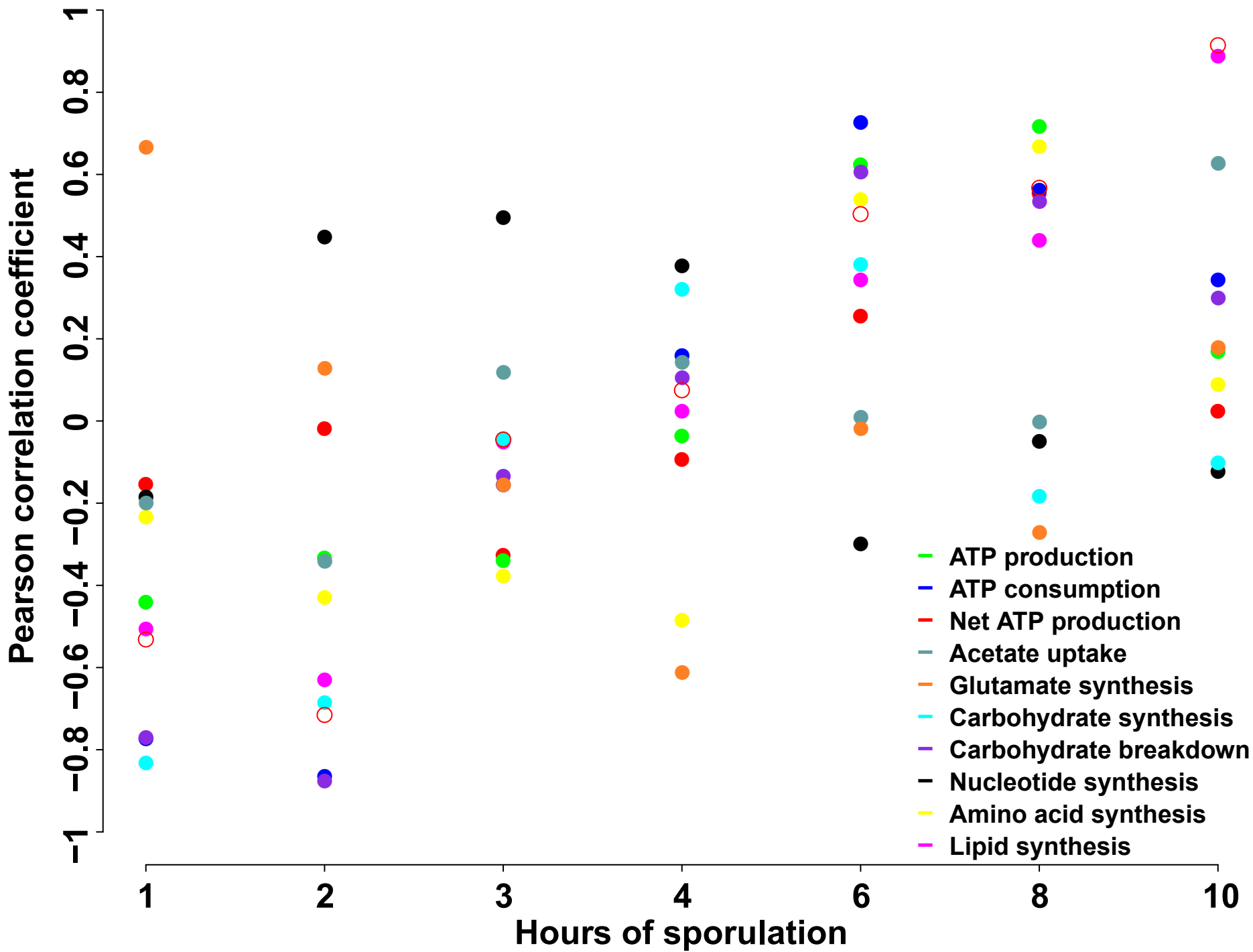

Supplement: Figure S3 — The use of microarray data to evaluate objective functions for the meiosis-specific network models. Reaction constraints are defined by gene expression from time-course Affymetrix data on SK1 sporulating cells at 1, 2, 3, 4, 6, 8, and 10 hours. The Pearson correlation is calculated between predicted fluxes and biochemical data on eight pathways when maximizing or minimizing each of the ten objective functions at each of the seven time points. The best objective function for each time point is the one with the maximum Pearson correlation coefficient. Five out of seven best objective functions at 1, 2, 3, 4, and 10 hours are consistent with those deduced from ribosome profiling. Close circle: maximization of an objective function; open circle: minimization of an objective function. Undefined correlation coefficients due to zero variance of predicted pathway fluxes are not shown in the figure. (PDF) [file pone.0063707.s003.pdf]

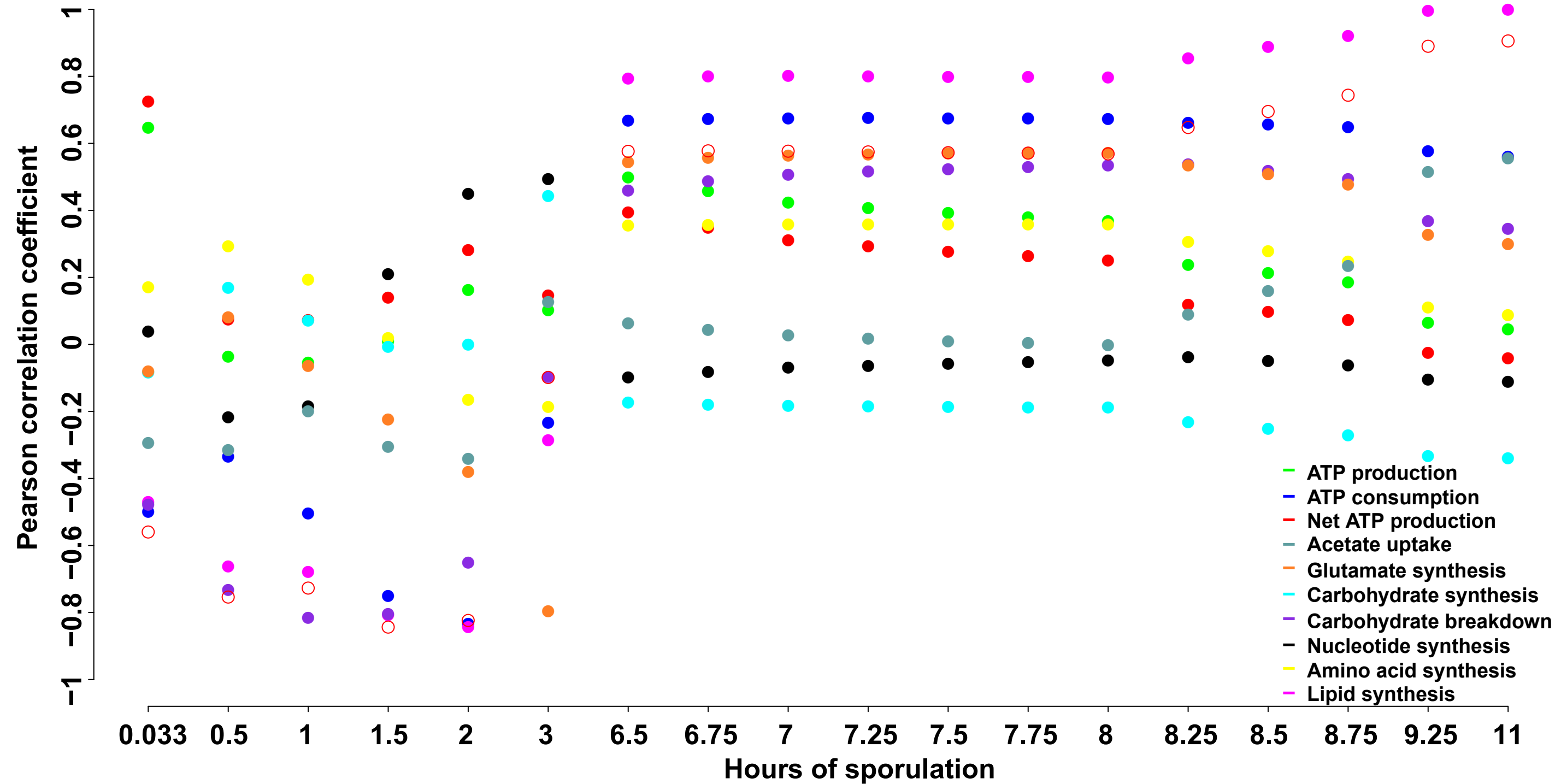

Supplement: Figure S4 — The use of uniform bounds to evaluate objective functions for the meiosis-specific network models. A uniform constraint of 1,000 is imposed for all reactions. The Pearson correlation is calculated between predicted fluxes and biochemical data on eight pathways when maximizing or minimizing each of the ten objective functions at each of the 18 time points. The best objective function for each time point is the one with the maximum Pearson correlation coefficient. Three out of 18 best objective functions at 0.033, 2, and 3 hours are consistent with those deduced from ribosome profiling. Close circle: maximization of an objective function; open circle: minimization of an objective function. Undefined correlation coefficients due to zero variance of predicted pathway fluxes are not shown in the figure. (PDF) [file pone.0063707.s004.pdf]

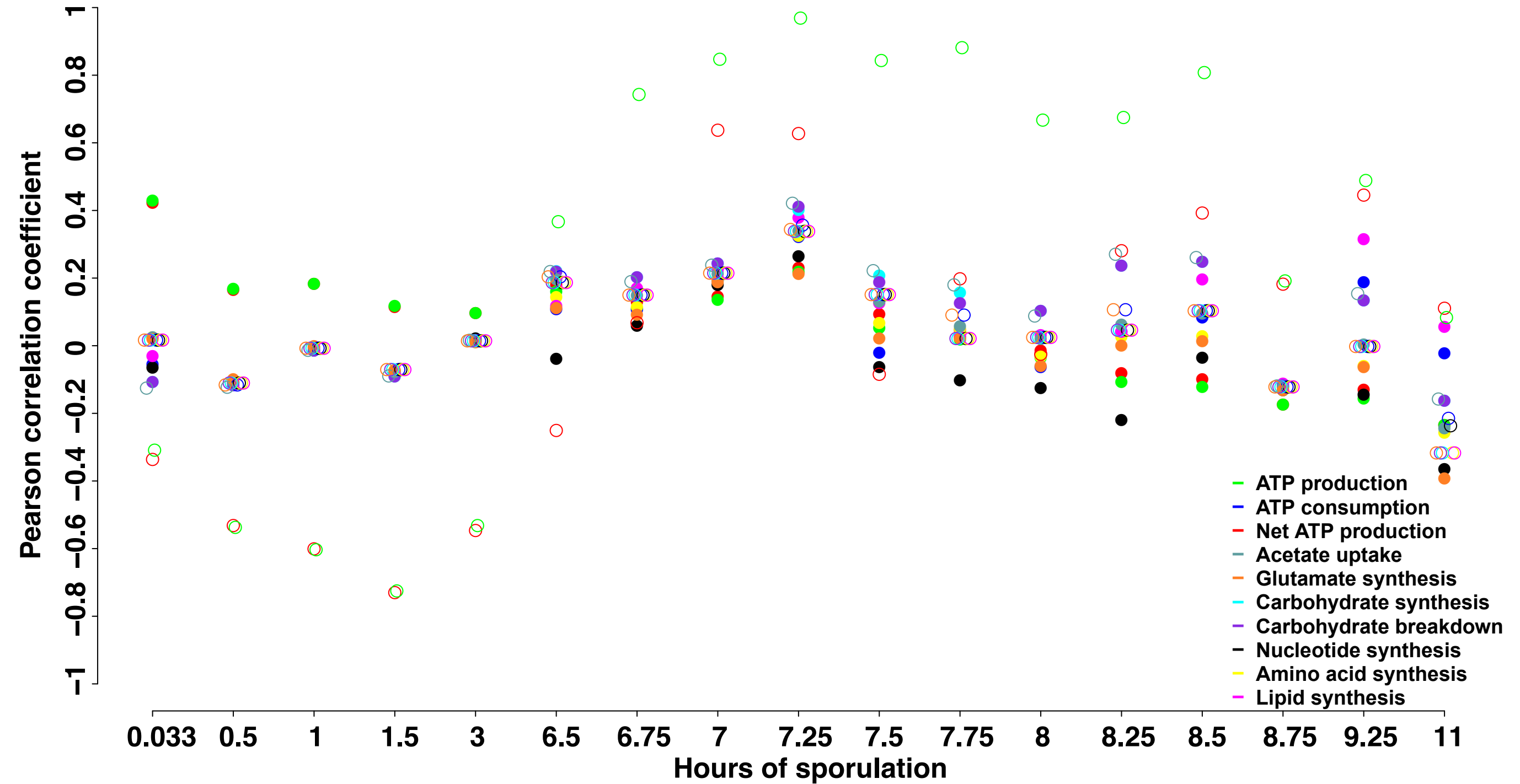

Supplement: Figure S5 — Evaluation of objective functions using the genome-scale network models. The Pearson correlation is calculated between predicted fluxes and biochemical data on eight pathways when maximizing or minimizing each of the ten objective functions at each of the 18 time points. The best objective function for each time point is the one with the maximum Pearson correlation coefficient. Close circle: maximization of an objective function; open circle: minimization of an objective function. Optimal solution does not exist regardless of objective functions at 2 hours of sporulation, thus does not shown in the figure. (PDF) [file pone.0063707.s005.pdf]
