# Supplementary material for: Characterization of the Metabolic Requirements in Yeast Meiosis
Source: PLoS One. 2013 May 8;8(5):e63707. doi: 10.1371/journal.pone.0063707 (PMC3650881; doi:10.1371/journal.pone.0063707)
Supplement: Figure S2 — The use of Spearman correlation to evaluate objective functions for the meiosis-specific network models. The Spearman correlation is calculated between predicted fluxes and biochemical data on eight pathways when maximizing or minimizing each of the ten objective functions at each of the 18 time points. The best objective function for each time point is the one with the maximum Spearman correlation coefficient. Eleven out of 18 best objective functions during 0.033–3 and 8.25–11 hours are consistent with those determined by the Pearson correlation as the first, second, or third ranked objective. Close circle: maximization of an objective function; open circle: minimization of an objective function. Undefined correlation coefficients due to zero variance of predicted pathway fluxes are not shown in the figure. (PDF) [file pone.0063707.s002.pdf]

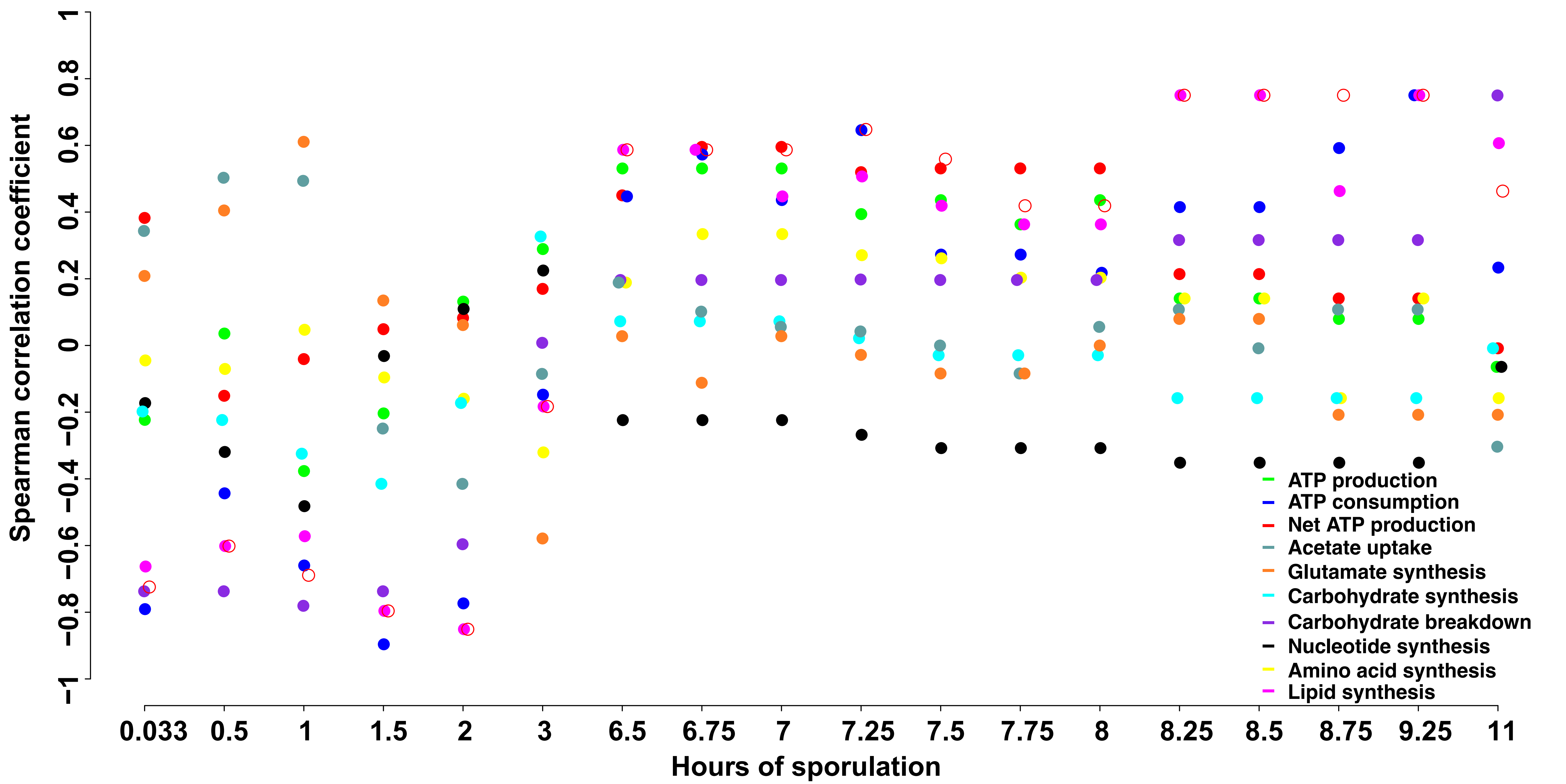

Spearman correlation coefficient
1
0.8
0.6
0.4
0.2
0
−0.2
−0.4
−0.6
−0.8
−1
0.033
0.5
1
1.5
2
3
6.5
6.75
7
7.25
7.5
7.75
8
8.25
8.5
8.75
9.25
11
Hours of sporulation
ATP production
ATP consumption
Net ATP production
Acetate uptake
Glutamate synthesis
Carbohydrate synthesis
Carbohydrate breakdown
Nucleotide synthesis
Amino acid synthesis
Lipid synthesis
