# Supplementary material for: Characterization of the Metabolic Requirements in Yeast Meiosis
Source: PLoS One. 2013 May 8;8(5):e63707. doi: 10.1371/journal.pone.0063707 (PMC3650881; doi:10.1371/journal.pone.0063707)
Supplement: Table S1 — Reactions in the yeast meiosis-specific metabolic network. (PDF) [file pone.0063707.s007.pdf]

Table S1. Reactions in the yeast meiosis-specific metabolic network.

Reaction	Metabolite[&]	Enzyme*	Objective function^	Pathway	Reference
Reactions occurring in the cytoplasm					
V1: Coenzyme A + Acetate + ATP => Acetyl-CoA +Pyrophosphate + AMP	Acetate, Acetyl-CoA	*ACS2*	Net ATP production (-1), ATP consumption (1)		[1,2]
V2: Oxaloacetate + Acetyl-CoA + H2O => Citrate + Coenzyme A	Oxaloacetate, Acetyl-CoA, Citrate	*CIT2*			[1,2,3,4]
V3: Citrate + H2O => Isocitrate	Citrate, Isocitrate	*ACO1*			[1,2,3]
V4: Isocitrate => Glyoxylate + Succinate	Isocitrate, Glyoxylate, Succinate	*ICL1*		Glyoxylate	[1,2,3]
V5: Glyoxylate + Acetyl-CoA => Malate	Glyoxylate, Acetyl-CoA, Malate	*MLS1*		Glyoxylate	[1,2,3]
V6: Malate + NAD => Oxaloacetate + NADH + H+	Malate, Oxaloacetate	*MDH2*	Net ATP production (3), ATP production (3)		[1,2,3]
V7: Oxaloacetate + GTP => CO2 + Phosphoenolpyruvate + GDP	Oxaloacetate, Phosphoenolpyruvate	*PCK1*	Net ATP production (-1), ATP consumption (1)	Gluconeogenesis	[2,5]
V8: Phosphoenolpyruvate + H2O => 2-Phosphoglycerate	Phosphoenolpyruvate, 2-Phosphoglycerate	*ENO1*		Gluconeogenesis	[2,3]
V9: 2-Phosphoglycerate => 3-Phosphoglycerate	2-Phosphoglycerate, 3-Phosphoglycerate	*GPM1*		Gluconeogenesis	[2,3]
V10: 3-Phosphoglycerate + ATP => 1,3-BIP Glycerate + ADP	3-Phosphoglycerate, 1,3-BIP Glycerate	*PGK1*	Net ATP production (-1), ATP consumption	Gluconeogenesis	[2,3,5]

			(1)		
V11: 1,3-BIP Glycerate + NADH + H+ => Glyceraldehyde-3-Phosphate + phosphate + NAD	1,3-BIP Glycerate, Glyceraldehyde-3-Phosphate	*TDH1* *TDH2* *TDH3* (or)	Net ATP production (-3), ATP consumption (3)	Gluconeogenesis	[2,3,6]
V12: Glyceraldehyde-3-Phosphate => Dihydroxy-acetone-phosphate	Glyceraldehyde-3-Phosphate,Dihydroxy-acetone-phosphate	*TPI1*		Gluconeogenesis	[2,3,6]
V13: Dihydroxy-Acetone-phosphate + Glyceraldehyde-3-phosphate => Fructose-1,6-bisphosphate	Dihydroxy-Acetone-phosphate, Glyceraldehyde-3-phosphate, Fructose-1,6-bisphosphate	*FBA1*		Gluconeogenesis	[2,3,6]
V14: Fructose-1,6-bisphosphate + H2O => Fructose-6-phosphate + phosphate	Fructose-1,6-bisphosphate, Fructose-6-phosphate	*FBP1*		Gluconeogenesis	[2,3,6]
V15: Fructose-6-phosphate => Glucose-6-phosphate	Fructose-6-phosphate, Glucose-6-phosphate	*PGI1*		Gluconeogenesis	[2,3,6]
V16: Glucose-6-phosphate => Glucose-1-phosphate	Glucose-6-phosphate, Glucose-1-phosphate	*PGM2*		Gluconeogenesis	[2,3]
V17: UDP-D-glucose + Glucose-6-phosphate => UDP + α,α-trehalose 6-phosphate<br><br>α,α-trehalose 6-phosphate + H2O => Trehalose + phosphate	Glucose-6-phosphate	*TPS1* *TPS2* *TPS3* *TLS1* (and)	Carbohydrate synthesis	Gluconeogenesis	[2,7,8]
V18: Glucose-1-phosphate + UTP => UDP-Glucose + 2 Pi<br><br>Glycogen $_{(n\ residues)}$ + UDP-Glucose => Glycogen $_{(n+1\ residues)}$ + UDP	Glucose-1-phosphate	*UGP1*	Carbohydrate synthesis	Gluconeogenesis	[2,8,9,10]
V19: a Glycogen + Phosphate => a Glycogen + Glucose-1-phosphate	Glucose-1-phosphate	*GPH1*	Carbohydrate breakdown	Glycogenolysis	[2,11,12]

V20: Glucose-1-phosphate => Glucose-6-phosphate	Glucose-1-phosphate, Glucose-6-phosphate	*PGM2*		Glycogenolysis	[1,2,3]
V21: Glucose-6-phosphate => Fructose-6-phosphate	Glucose-6-phosphate, Fructose-6-phosphate	*PGI1*		Glycogenolysis	[2,3,6]
V22: Fructose-6-phosphate +ATP => Fructose-1,6-bisphosphate + ADP + H^+	Fructose-6-phosphate , Fructose-1,6-bisphosphate	*PFK1* *PFK2* (and)	Net ATP production (-1), ATP consumption (1)	Glycogenolysis	[2,3,6]
V23: Fructose-1,6-bisphosphate => Dihydroxy-Acetone-phosphate + Glyceraldehyde-3-phosphate	Fructose-1,6-bisphosphate, Dihydroxy-Acetone-phosphate, Glyceraldehyde-3-phosphate	*FBA1*		Glycogenolysis	[2,3,6]
V24: Dihydroxy-Acetone-phosphate => Glyceraldehyde-3-phosphate	Dihydroxy-Acetone-phosphate, Glyceraldehyde-3-phosphate	*TPI1*		Glycogenolysis	[2,3,6]
V25: Glyceraldehyde-3-phosphate + Phosphate + NAD => 1,3-BIP Glycerate + NADH + H^+	Glyceraldehyde-3-phosphate, 1,3-BIP Glycerate	*TDH1* *TDH2* *TDH3* (or)	Net ATP production (3), ATP production (3)	Glycogenolysis	[2,3,6]
V26: 1,3-BIP Glycerate + ADP => 3-Phosphoglycerate + ATP	1,3-BIP Glycerate, 3-Phosphoglycerate	*PGK1*	Net ATP production (1), ATP production (1)	Glycogenolysis	[2,3,6]
V27: 3-Phosphoglycerate => 2-Phosphoglycerate	3-Phosphoglycerate, 2-Phosphoglycerate	*GPM1*		Glycogenolysis	[2,3,6]
V28: 2-Phosphoglycerate => Phosphoenolpyruvate + H2O	2-Phosphoglycerate, Phosphoenolpyruvate	*ENO1*		Glycogenolysis	[2,3,6]
V29: Phosphoenolpyruvate + ADP + H+ => Pyruvate + ATP	Phosphoenolpyruvate, Pyruvate	*PYK2*	Net ATP production (1), ATP production (1)	Glycogenolysis	[2,3,6]

V30: HCO3- + Pyruvate + ATP => Phosphate + Oxaloacetate + ADP	Pyruvate, Oxaloacetate	*PYC1* *PYC2* (or)	Net ATP production (-1), ATP consumption (1)	Glycogenolysis	[1,2]
V31: Fumarate + H2O => Malate	Fumarate, Malate	*FUM1*			[2,3,5]
V32: Isocitrate + NADP => Oxoglutarate + CO2 + NADPH	Isocitrate, Oxoglutarate	*IDP2*	Net ATP production (3), ATP production (3)	Glutamate	[2,3]
V33: Oxoglutarate + NH3 + NADPH + H+ => L-Glutamate + H2O + NADP	Oxoglutarate	*GDH3*	Net ATP production (-3), ATP consumption (3), Glutamate synthesis	Glutamate	[2,3,8]
Reactions occurring in the mitochondria					
V34: Coenzyme A [m] + NAD + Pyruvate [m] => Acetyl-CoA [m] + CO2 + NADH	Pyruvate [m], Acetyl-CoA [m]	*PDB1* *PDA1* (and)		Glycogenolysis	[2,3,6]
V35: Oxaloacetate [m] + Acetyl-CoA [m] + H2O => Citrate [m]+ Coenzyme A [m]	Oxaloacetate [m], Acetyl-CoA [m], Citrate [m]	*CIT1*		TCA	[1,2,3,4]
V36: Citrate [m] + H2O => Isocitrate [m]	Citrate [m], Isocitrate [m]	*ACO1*		TCA	[1,2,3]
V37: NAD + Isocitrate [m] => Oxoglutarate [m] + NADH + CO2	Isocitrate [m], Oxoglutarate [m]	*IDH1* *IDH2* (and)	Net ATP production (3), ATP production (3)	TCA	[1,2,3]
V38: Oxoglutarate [m] + Coenzyme A [m] + NAD => Succinyl-CoA [m] + CO2 + NADH	Oxoglutarate [m], Succinyl-CoA [m]	*LPD1* *KGD1* *KGD2* (and)	Net ATP production (3), ATP	TCA	[2,3,5]

			production (3)		
V39: Succinyl-CoA [m] + GDP + Phosphate <=> Succinate [m] + Coenzyme A [m] + GTP	Succinyl-CoA [m], Succinate [m]	*LSC1* *LSC2* (and)	Net ATP production (1), ATP production (1)	TCA	[2,3,5]
V40: Succinate [m] + FAD => Fumarate [m] + FADH2	Succinate [m], Fumarate [m]	*SDH1* *SDH2* *SDH3* *SDH4* (and)	Net ATP production (2), ATP production (2)	TCA	[1,2,3]
V41: Fumarate [m] + H2O => Malate [m]	Fumarate [m], Malate [m]	*FUM1*		TCA	[1,2,3]
V42: Malate [m] + NAD => Oxaloacetate [m] + NADH + H^+	Malate [m], Oxaloacetate [m]	*MDH1*	Net ATP production (3), ATP production (3)	TCA	[1,2,3]
Transport reactions					
V43: Acetate [e] => Acetate	Acetate	*ADY2*	Acetate uptake		[2,13,14]
V44: Acetyl-CoA+ Carnitine => CoA + O-Acetylcarnitine<br><br>O-Acetylcarnitine + CoA => Acetyl-CoA [m] + Carnitine [$]	Acetyl-CoA, Acetyl-CoA [m]	*CAT2* *YAT1* *YAT2* (avg)			[2,15]
V45: Oxaloacetate [m] => Oxaloacetate	Oxaloacetate [m], Oxaloacetate	*OAC1*			[2,16,17]
V46: Oxaloacetate => Oxaloacetate [m]	Oxaloacetate, Oxaloacetate [m]	*OAC1*			[2,16,17]
V47: Citrate => Citrate [m]	Citrate, Citrate [m]	*CTP1*			[2,18]
V48: Isocitrate [m] => Isocitrate	Isocitrate, Isocitrate [m]	*CTP1*			[2,18]
V49: Fumarate [m] + Succinate => Fumarate + Succinate [m] [#]	Fumarate [m], Succinate, Fumarate, Succinate [m]	*SFC1*			[2,17,19]

V50: Succinate + Phosphate [m] => Succinate [m] + Phosphate	Succinate [m], Succinate	*DIC1*			[2,17,19]
V51: Oxoglutarate [m] => Oxoglutarate	Oxoglutarate [m], Oxoglutarate	*ODC1*		Glutamate	[2,20]
V52: Pyruvate => Pyruvate [m]	Pyruvate, Pyruvate [m]	*YIA6*		Glycogenolysis	[2,21,22]
Reactions that produce nucleotides, amino acids, and lipids					
V53: Glucose-6-phosphate + NADP => D-6-phospho-glucono-δ-lactone + NADPH + H^+	Glucose-6-phosphate	*ZWF1*	Net ATP production (3), ATP production (3), Nucleotide synthesis	Nucleotide	[2,23,24]
V54: 3-Phosphoglycerate + NAD+ => 3-Phosphohydroxypyruvate + H + NADH	3-Phosphoglycerate	*SER3* *SER33* (or)	Net ATP production (3), ATP production (3), Amino acid synthesis	Amino acid	[2,23,24]
V55: Oxaloacetate + L-glutamate => 2-oxoglutarate + L-aspartate	Oxaloacetate	*AAT2*	Amino acid synthesis	Amino acid	[2,23,24]
V56: Pyruvate [m] + NADPH + H+ + glutamate => CO2 + NADP+ + H2O + Alpha-ketoglutarate + valine	Pyruvate [m]	*ILV2* *ILV6* (and)	Amino acid synthesis	Amino acid	[2,23,24]
V57: Oxaloacetate [m] + L-Glutamate => 2-Oxoglutarate + L-Aspartate	Oxaloacetate [m]	*AAT1*	Amino acid synthesis	Amino acid	[2,23,24]
V58: Phosphoenolpyruvate + Skm5p => 3Psme + phosphate	Phosphoenolpyruvate	*ARO1*	Amino acid synthesis	Amino acid	[2,23,24]
V59: BCCP-biotin-CO2 + Acetyl-CoA + ATP => Malonyl-CoA + BCCP-	Acetyl-CoA	*ACC1*	Net ATP production (-1),	Lipid	[2,23,24]

biotin + phosphate + ADP			ATP consumption (1), Lipid synthesis		
V60: BCCP-biotin-CO2 + Acetyl-CoA [m]+ ATP => Malonyl-CoA + BCCP-biotin + phosphate + ADP	Acetyl-CoA [m]	*HFA1*	Net ATP production (-1), ATP consumption (1), Lipid synthesis	Lipid	[2,23,24]
V61: Dihydroxy-Acetone-phosphate + NADH => Glycerol 3 P + NAD	Dihydroxy-Acetone-phosphate	*GPD1*	Net ATP production (-3), ATP consumption (3), Lipid synthesis	Lipid	[2,23,24]
V62: Glucose-6-phosphate => Mi1p-D	Glucose-6-phosphate	*INO1*	Lipid synthesis	Lipid	[2,24]

& All metabolites are located in the cytoplasm except those labeled with [m] (mitochondrial metabolites) or [e] (extracellular metabolites).

* The "and" logic implies protein complexes or multi-subunit proteins where each enzyme is required to catalyze a reaction; the minimum expression value of all enzymes is used to define the upper bound of reaction constraints. The "or" logic implies isozymes where each enzyme can individually catalyze a reaction; the maximum expression value of all enzymes is used to define the upper bound of reaction constraints.

^ Each objective function is defined by $C^T V$, where V is a vector of reaction fluxes and C is a vector of zeros with a value of one at the position of reactions of interest, as stated in the column. The exceptions are three ATP objectives: positive numbers of yielded ATPs are coefficients for reactions participating in ATP production, positive numbers of used ATPs are coefficient for reactions participating in ATP consumption, and positive numbers of yielded ATPs and negative numbers of used ATPs are entered as coefficients for the objective function of net ATP production. The ATP coefficients are in parentheses immediately following the name of the ATP-related objective functions. Other energy molecules are converted to the ATP unit: 1NADH=3ATP, 1NADPH=3ATP, 1FADH2=2ATP, 1GTP=1ATP.

$ This reaction represents three independent reactions catalyzed by three enzymes, thus the average expression value of the three enzymes is used to define the upper bound of reaction constraints [15].

The flux of this reaction is set to 0 because *SFC1* encodes an antiporter, which transports cytoplasmic succinate into mitochondria in exchange for fumarate [19].

REFERENCES

1. Wang Q, Zhang Y, Yang C, Xiong H, Lin Y, et al. (2010) Acetylation of metabolic enzymes coordinates carbon source utilization and metabolic flux. Science 327: 1004-1007.
2. Engel SR, Balakrishnan R, Binkley G, Christie KR, Costanzo MC, et al. (2010) Saccharomyces Genome Database provides mutant phenotype data. Nucleic Acids Res 38: D433-436.
3. Kanehisa M, Goto S, Furumichi M, Tanabe M, Hirakawa M (2010) KEGG for representation and analysis of molecular networks involving diseases and drugs. Nucleic Acids Res 38: D355-360.
4. Kim KS, Rosenkrantz MS, Guarente L (1986) Saccharomyces cerevisiae contains two functional citrate synthase genes. Mol Cell Biol 6: 1936-1942.
5. Blank LM, Kuepfer L, Sauer U (2005) Large-scale 13C-flux analysis reveals mechanistic principles of metabolic network robustness to null mutations in yeast. Genome Biol 6: R49.
6. Zhao S, Xu W, Jiang W, Yu W, Lin Y, et al. (2010) Regulation of cellular metabolism by protein lysine acetylation. Science 327: 1000-1004.
7. Vandercammen A, Francois J, Hers HG (1989) Characterization of trehalose-6-phosphate synthase and trehalose-6-phosphate phosphatase of Saccharomyces cerevisiae. Eur J Biochem 182: 613-620.
8. Walker GM (1998) Yeast physiology and biotechnology. West Sussex: John Wiley & Sons Ltd.
9. Cheng C, Mu J, Farkas I, Huang D, Goebl MG, et al. (1995) Requirement of the self-glucosylating initiator proteins Glg1p and Glg2p for glycogen accumulation in Saccharomyces cerevisiae. Mol Cell Biol 15: 6632-6640.
10. Daran JM, Dallies N, Thines-Sempoux D, Paquet V, Francois J (1995) Genetic and biochemical characterization of the UGP1 gene encoding the UDP-glucose pyrophosphorylase from Saccharomyces cerevisiae. Eur J Biochem 233: 520-530.
11. Rath VL, Hwang PK, Fletterick RJ (1992) Purification and crystallization of glycogen phosphorylase from Saccharomyces cerevisiae. J Mol Biol 225: 1027-1034.
12. Hwang PK, Tugendreich S, Fletterick RJ (1989) Molecular analysis of GPH1, the gene encoding glycogen phosphorylase in Saccharomyces cerevisiae. Mol Cell Biol 9: 1659-1666.
13. Casal M, Cardoso H, Leao C (1996) Mechanisms regulating the transport of acetic acid in Saccharomyces cerevisiae. Microbiology 142 (Pt 6): 1385-1390.
14. Paiva S, Devaux F, Barbosa S, Jacq C, Casal M (2004) Ady2p is essential for the acetate permease activity in the yeast Saccharomyces cerevisiae. Yeast 21: 201-210.
15. Swiegers JH, Dippenaar N, Pretorius IS, Bauer FF (2001) Carnitine-dependent metabolic activities in Saccharomyces cerevisiae: three carnitine acetyltransferases are essential in a carnitine-dependent strain. Yeast 18: 585-595.
16. Palmieri L, Vozza A, Agrimi G, De Marco V, Runswick MJ, et al. (1999) Identification of the yeast mitochondrial transporter for oxaloacetate and sulfate. J Biol Chem 274: 22184-22190.
17. Palmieri L, Lasorsa FM, Vozza A, Agrimi G, Fiermonte G, et al. (2000) Identification and functions of new transporters in yeast mitochondria. Biochim Biophys Acta 1459: 363-369.
18. Kaplan RS, Mayor JA, Gremse DA, Wood DO (1995) High level expression and characterization of the mitochondrial citrate transport protein from the yeast Saccharomyces cerevisiae. J Biol Chem 270: 4108-4114.
19. Palmieri L, Lasorsa FM, De Palma A, Palmieri F, Runswick MJ, et al. (1997) Identification of the yeast ACR1 gene product as a succinate-fumarate transporter essential for growth on ethanol or acetate. FEBS Lett 417: 114-118.
20. Palmieri L, Agrimi G, Runswick MJ, Fearnley IM, Palmieri F, et al. (2001) Identification in Saccharomyces cerevisiae of two isoforms of a novel mitochondrial transporter for 2-oxoadipate and 2-oxoglutarate. J Biol Chem 276: 1916-1922.

21. Hildyard JC, Halestrap AP (2003) Identification of the mitochondrial pyruvate carrier in Saccharomyces cerevisiae. Biochem J 374: 607-611.
22. Sugden MC, Holness MJ (2003) Trials, tribulations and finally, a transporter: the identification of the mitochondrial pyruvate transporter. Biochem J 374: e1-2.
23. Cortassa S, Aon JC, Aon MA (1995) Fluxes of carbon, phosphorylation, and redox intermediates during growth of saccharomyces cerevisiae on different carbon sources. Biotechnol Bioeng 47: 193-208.
24. Nelson DL, Cox MM (2008) Lehninger Principles of Biochemistry: Freeman, W. H. & Company
