# Supplementary material for: Characterization of the Metabolic Requirements in Yeast Meiosis
Source: PLoS One. 2013 May 8;8(5):e63707. doi: 10.1371/journal.pone.0063707 (PMC3650881; doi:10.1371/journal.pone.0063707)
Supplement: Table S3 — Biochemical data on metabolic pathways and macromolecule syntheses during yeast meiosis. (PDF) [file pone.0063707.s009.pdf]

Table S3. Biochemical data on metabolic pathways and macromolecule syntheses during yeast meiosis[&].

Hours	Scaled SK1 hours	Measurements	Activities	Scaled activities
Glutamate formation: *the proportion of 1,2,3,4-^{13}C-labeled glutamate is measured by NMR spectroscopy (Figure 4 of [1]). The S41 strain reaches the steady state level of ascus formation in 36 hours (Figure 4B of [2]).*				
0	0	0	0	0
1	0.33	10	10	1
2	0.67	20	10	1
4	1.33	30	10	1
8	2.66	33	3	0.3
TCA cycle: *the percentage of oxygen consumed per minute is measured by an oxygen monitor (Figure 1 of [3]). The AP1 strain reaches the steady state level of ascus formation in 24 hours (Figure 1 of [3]).*				
0	0	20	20	0.8
2	1	25	25	1
4	2	20	20	0.8
6	3	15	15	0.6
8	4	10	10	0.4
10	5	8	8	0.32
12	6	6	6	0.24
14	7	4	4	0.16
16	8	3	3	0.12
Gluconeogenesis: *mg carbohydrate /10^8 cells is measured by the anthrone procedure (Figure 2A of [4]). The SK1 strain reaches the steady state level of ascus formation in 12 hours (Figure 1 of [4]).*				
0	0	0.4	0	0
2	2	0.9	0.5	0.45
4	4	2	1.1	1
6	6	2.2	0.2	0.18
Glycogenolysis: *glycogenolytic enzyme activity is measured (Figure 2 of [5]). The AP1 strain reaches the steady state level of ascus formation in 24 hours (Figure 2 of [5]).*				
0	0	0	0	0
4	2	0	0	0
6	3	0	0	0
8	4	8	8	0.15
9	4.5	12	12	0.23
10	5	18	18	0.34
12	6	22	22	0.42
14	7	40	40	0.75
16	8	50	50	0.94
18	9	52	52	0.98
20	10	53	53	1
24	12	50	50	0.94
DNA: *percentage of DNA content is measured by the diaminobenzoic acid fluorimetric procedure (Figure 2A of [3]). The AP1 strain reaches the steady state level of ascus formation in 24 hours (Figure 1 of [3]).*				
0	0	0	0	0
1	0.5	5	5	0.25

2	1	9	4	0.2
3	1.5	20	11	0.55
4	2	40	20	1
5	2.5	60	20	1
8	4	80	20	1
RNA: *^{3}H incorporated into RNA is measured (Figure 3A of [3]). The AP1 strain reaches the steady state level of ascus formation in 24 hours (Figure 1 of [3]).*				
0	0	40	40	0.29
0.5	0.25	50	50	0.36
1.5	0.75	65	65	0.46
2	1	80	80	0.57
3	1.5	135	135	0.96
4	2	140	140	1
5	2.5	120	120	0.86
6	3	100	100	0.71
7	3.5	90	90	0.64
8	4	70	70	0.5
9	4.5	60	60	0.43
10	5	50	50	0.36
11	5.5	40	40	0.29
Protein: *^{35}S incorporated into protein is measured (Figure 3A of [3]). The AP1 strain reaches the steady state level of ascus formation in 24 hours (Figure 1 of [3]).*				
0	0	90	90	0.56
1	0.5	120	120	0.75
1.5	0.75	150	150	0.94
2	1	160	160	1
3	1.5	155	155	0.97
3.5	1.75	150	150	0.94
4	2	120	120	0.75
4.5	2.25	110	110	0.69
5	2.5	90	90	0.56
Lipid: *^{14}C-acetate incorporated into lipids is measured (Figure 2A of [6]). The Y-185 strain reaches the steady state level of ascus formation in 30 hours (Figure 1 of [6]).*				
0	0	0	0	0
2.5	1	3	3	0.46
5	2	5	2	0.31
6	2.4	6.5	1.5	0.23
8	3.2	11	4.5	0.69
10	4	15.5	4.5	0.69
11	4.4	16	0.5	0.08
12	4.8	17	1	0.15
14	5.6	18	1	0.15
19	7.6	19	1	0.15
20	8	20	1	0.15
22	8.8	22.5	2.5	0.38
24	9.6	29	6.5	1

30	12	33	4	0.62

[&] The scaled SK1 hours and the scaled activities are plotted in Figure 2.

REFERENCES

1. Dickinson JR, Dawes IW, Boyd AS, Baxter RL (1983) 13C NMR studies of acetate metabolism during sporulation of Saccharomyces cerevisiae. Proc Natl Acad Sci U S A 80: 5847-5851.
2. Esposito MS, Esposito RE, Arnaud M, Halvorson HO (1969) Acetate utilization and macromolecular synthesis during sporulation of yeast. J Bacteriol 100: 180-186.
3. Hopper AK, Magee PT, Welch SK, Friedman M, Hall BD (1974) Macromolecule synthesis and breakdown in relation to sporulation and meiosis in yeast. J Bacteriol 119: 619-628.
4. Kane SM, Roth R (1974) Carbohydrate metabolism during ascospore development in yeast. J Bacteriol 118: 8-14.
5. Colonna WJ, Magee PT (1978) Glycogenolytic enzymes in sporulating yeast. J Bacteriol 134: 844-853.
6. Henry SA, Halvorson HO (1973) Lipid synthesis during sporulation of Saccharomyces cerevisiae. J Bacteriol 114: 1158-1163.
