# Supplementary material for: Characterization of the Metabolic Requirements in Yeast Meiosis
Source: PLoS One. 2013 May 8;8(5):e63707. doi: 10.1371/journal.pone.0063707 (PMC3650881; doi:10.1371/journal.pone.0063707)
Supplement: Table S4 — Interpolated biochemical data on metabolic pathways and macromolecule syntheses at 18 time points. (PDF) [file pone.0063707.s010.pdf]

Table S4. Interpolated biochemical data on metabolic pathways and macromolecule syntheses at 18 time points[&].

**Hours of sporulation**	**Glutamate formation**	**TCA cycle**	**Glyoxylate cycle**	**Gluconeogenesis**	**Glycogenolysis**	**Nucleotide**	**Amino acid**	**Lipid**
0.033	*0.10*	*0.80*	*0.10*	*0.01*	*0.00*	*0.30*	*0.57*	*0.02*
0.5	*1.00*	*0.90*	*1.00*	*0.11*	*0.00*	*0.41*	**0.75**	*0.23*
1	*1.00*	**1.00**	*1.00*	*0.23*	*0.00*	**0.57**	**1.00**	**0.46**
1.5	*0.91*	*0.90*	*0.91*	*0.34*	*0.00*	**0.96**	**0.97**	*0.39*
2	*0.64*	**0.80**	*0.64*	**0.45**	**0.00**	**1.00**	**0.75**	**0.31**
3	0	**0.60**	*0.73*	*0.73*	**0.00**	*1.00*	0	*0.58*
6.5	0	*0.20*	0	0	*0.59*	0	0	*0.15*
6.75	0	*0.18*	0	0	*0.67*	0	0	*0.15*
7	0	**0.16**	0	0	**0.75**	0	0	*0.15*
7.25	0	*0.15*	0	0	*0.80*	0	0	*0.15*
7.5	0	*0.14*	0	0	*0.85*	0	0	*0.15*
7.75	0	*0.13*	0	0	*0.89*	0	0	*0.15*
8	0	**0.12**	0	0	**0.94**	0	0	**0.15**
8.25	0	0	0	0	*0.95*	0	0	*0.22*
8.5	0	0	0	0	*0.96*	0	0	*0.29*
8.75	0	0	0	0	*0.97*	0	0	*0.37*
9.25	0	0	0	0	*0.99*	0	0	*0.73*
11	0	0	0	0	*0.97*	0	0	*0.78*

[&] The scaled biochemical data are described in Table S3. Values obtained directly from the scaled data in Table S3 are in bold. Values obtained from linear interpolation of the scaled data in Table S3 are in italic. Missing values equal 0. Values for the glyoxylate cycle are the maximum of glutamate formation and gluconeogenesis at each time point. Values for nucleotide synthesis are the maximum of DNA and RNA levels at each time point.
